# Computing and visualising intra‐voxel orientation‐specific relaxation–diffusion features in the human brain

**DOI:** 10.1002/hbm.25224

**Published:** 2020-10-06

**Authors:** João P. de Almeida Martins, Chantal M. W. Tax, Alexis Reymbaut, Filip Szczepankiewicz, Maxime Chamberland, Derek K. Jones, Daniel Topgaard

**Affiliations:** ^1^ Division of Physical Chemistry, Department of Chemistry Lund University Lund Sweden; ^2^ Random Walk Imaging AB Lund Sweden; ^3^ Cardiff University Brain Research Imaging Centre (CUBRIC), Cardiff University Cardiff UK; ^4^ University Medical Center Utrecht, Utrecht University Utrecht The Netherlands; ^5^ Department of Clinical Sciences Lund University Lund Sweden; ^6^ Harvard Medical School Boston Massachusetts USA; ^7^ Radiology, Brigham and Women's Hospital Boston Massachusetts USA; ^8^ Mary MacKillop Institute for Health Research, Australian Catholic University Melbourne Australia

**Keywords:** diffusion MRI, fibre ODF, fibre‐specific metrics, partial volume effects, tensor‐valued diffusion encoding, white matter

## Abstract

Diffusion MRI techniques are used widely to study the characteristics of the human brain connectome in vivo. However, to resolve and characterise white matter (WM) fibres in heterogeneous MRI voxels remains a challenging problem typically approached with signal models that rely on prior information and constraints. We have recently introduced a 5D relaxation–diffusion correlation framework wherein multidimensional diffusion encoding strategies are used to acquire data at multiple echo‐times to increase the amount of information encoded into the signal and ease the constraints needed for signal inversion. Nonparametric Monte Carlo inversion of the resulting datasets yields 5D relaxation–diffusion distributions where contributions from different sub‐voxel tissue environments are separated with minimal assumptions on their microscopic properties. Here, we build on the 5D correlation approach to derive fibre‐specific metrics that can be mapped throughout the imaged brain volume. Distribution components ascribed to fibrous tissues are resolved, and subsequently mapped to a dense mesh of overlapping orientation bins to define a smooth orientation distribution function (ODF). Moreover, relaxation and diffusion measures are correlated to each independent ODF coordinate, thereby allowing the estimation of orientation‐specific relaxation rates and diffusivities. The proposed method is tested on a healthy volunteer, where the estimated ODFs were observed to capture major WM tracts, resolve fibre crossings, and, more importantly, inform on the relaxation and diffusion features along with distinct fibre bundles. If combined with fibre‐tracking algorithms, the methodology presented in this work has potential for increasing the depth of characterisation of microstructural properties along individual WM pathways.

## Graphical abstract

Diffusion MRI techniques designed to resolve fibre crossings within a given white matter (WM) voxel typically assume that the voxel‐level microstructural features can be represented by a single signal response function; this precludes the investigation of microscopic differences between the sub‐voxel fibre populations. In this work, we build on a recently introduced 5D relaxation–diffusion correlation MRI framework and present an analysis protocol for deriving and visualising metrics informing on the relaxation rates and diffusivities of distinct fibres. Experiments on a healthy volunteer demonstrate that the presented approach can capture crossings between distinct WM tracts of the human brain and inform on their individual relaxation–diffusion properties.

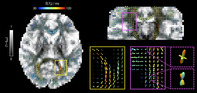

## 1 INTRODUCTION

The advent of diffusion MRI techniques, which can probe structures at much smaller scales than the imaging resolution by virtue of sensing the random motion of water molecules, has undoubtedly increased the interest in studying white matter (WM) in the living brain. The possibility of deriving quantitative features sensitive to tissue microstructure (Basser & Pierpaoli, 1996; Le Bihan, 1995), and to virtually reconstruct brain connections with fibre‐tracking algorithms (Basser, Pajevic, Pierpaoli, Duda, & Aldroubi, 2000; Mori, Crain, Chacko, & Van Zijl, 1999) led to the quick adoption of diffusion MRI in many clinical research applications (Barnea‐Goraly et al., 2004; Lebel, Walker, Leemans, Phillips, & Beaulieu, 2008; Lim et al., 1999; Werring, Clark, Barker, Thompson, & Miller, 1999). More recently, tractometry techniques have been developed to tease out WM pathways and characterise their individual tissue microstructure by mapping sets of diffusion‐derived parameters along with the extracted tracks (Bells et al., 2011; Chamberland et al., 2019; De Santis, Drakesmith, Bells, Assaf, & Jones, 2014; Rheault, Houde, & Descoteaux, 2017; Yeatman, Dougherty, Myall, Wandell, & Feldman, 2012). Fibre‐tracking techniques typically rely on the estimation of a fibre Orientation Distribution Function (ODF) per voxel, which is a function on the unit sphere aiming to represent the relative number of fibres along each direction (Dell'Acqua & Tournier, 2019; Tournier, 2019). It should be noted that the fibre ODF is distinct from the orientation distribution of the diffusion signal, and its extraction relies on assessing how tissue microstructure influences the measured MRI signal.

Diffusion MRI studies of WM commonly assume that voxel‐level microstructural features can be adequately represented by a single canonical signal response function (Dell'Acqua & Tournier, 2019; Novikov, Fieremans, Jespersen, & Kiselev, 2019). Under this assumption, the measured signal is written as the convolution between the fibre ODF and a kernel describing the signal response of a set of fibres with a common orientation. The simultaneous unconstrained estimation of the ODF and the microstructural kernel, however, has proven to be notoriously challenging for the diffusion MRI protocols typically used for in vivo research studies (Jelescu, Veraart, Fieremans, & Novikov, 2016). The complexity of this problem is commonly reduced by imposing a set of priors and constraints. Spherical deconvolution of the diffusion MRI signal (Anderson, 2005; Dell'Acqua et al., 2007; Dell'Acqua & Tournier, 2019; Jian & Vemuri, 2007; Tournier, Calamante, & Connelly, 2007; Tournier, Calamante, Gadian, & Connelly, 2004), for example, determines an empirical kernel for the whole brain representing the signal response of a single‐fibre population and subsequently solves for the ODF. For voxels containing not only WM but also unknown amounts of grey matter (GM), cerebrospinal fluid (CSF), or pathological tissue, this approach can yield biased ODF estimates.

Multi‐tissue spherical deconvolution (Jeurissen, Tournier, Dhollander, Connelly, & Sijbers, 2014) has been proposed to simultaneously resolve sub‐voxel tissue fractions and the fibre ODF. While this technique can be used to separate the sub‐voxel signal contributions from WM, GM, and CSF, it still assumes a single kernel for all voxels of a given tissue type, which needs to be calibrated a priori (Tax, Jeurissen, Vos, Viergever, & Leemans, 2014). Inaccuracies of the calibrated kernels can further bias the estimated fractions and fibre ODFs (Guo et al., 2019; Parker et al., 2013). Alternatively, the voxel‐wise kernel can be estimated by first factoring out the ODF through the computation of rotational invariants, and then fitting the data to signal models that set a pre‐defined number of microscopic environments with potentially constrained diffusion properties (Kaden, Kelm, Carson, Does, & Alexander, 2016; Novikov et al., 2019; Novikov, Veraart, Jelescu, & Fieremans, 2018). However, different fibre populations within a voxel likely exhibit different microstructural properties (Aboitiz, Scheibel, Fisher, & Zaidel, 1992; De Santis, Assaf, Jeurissen, Jones, & Roebroeck, 2016; Howard et al., 2019; Scherrer et al., 2016), which cannot be reflected with a single voxel‐wise kernel. It should furthermore be noted that differences in the transverse relaxation time *T*_2_ between distinct tissue types are often ignored, which can further bias the quantification of tissue fractions with a single fibre response kernel (Tax, Kleban, Barakovic, Chamberland, & Jones, 2020). The possible existence of a variation of *T*_2_ in anisotropic structures with respect to the orientation of the main magnetic field ***B***_0_ (Lindblom, Wennerström, & Arvidson, 1977), well known in studies of cartilage structure (Henkelman, Stanisz, Kim, & Bronskill, 1994) and more recently reported in in vivo human WM studies (Gil et al., 2016; Knight, Wood, Couthard, & Kauppinen, 2015; McKinnon & Jensen, 2019; Tax et al., 2020), would introduce an additional *T*_2_ dispersion and further complicate the quantification of sub‐voxel signal fractions. The possible existence of *T*_2_ differences between distinct fibre bundles has motivated the recent development of methods allowing for the measurement of fibre‐specific estimates of the transverse relaxation time (de Almeida Martins & Topgaard, 2018; Ning, Gagoski, Szczepankiewicz, Westin, & Rathi, 2020; Schiavi et al., 2019).

Inspired by multidimensional solid‐state NMR methodology (Schmidt‐Rohr & Spiess, 1994; Topgaard, 2017), we have introduced a framework to quantify the composition of each voxel with joint distributions of effective transverse relaxation rates *R*_2_ = 1/*T*_2_ and apparent diffusion tensors **D** (de Almeida Martins et al., 2020; de Almeida Martins & Topgaard, 2018). Specifically, the inclusion of diffusion MRI data measured with multidimensional diffusion encoding schemes (Topgaard, 2017) and different echo times was observed to alleviate the constraints needed to resolve sub‐voxel tissue heterogeneity (de Almeida Martins et al., 2020). Capitalising on these acquisitions, we quantified sub‐voxel compositions using 5D discrete *R*_2_‐**D** distributions retrieved from the data using a nonparametric Monte Carlo inversion procedure. However, visualising the retrieved sub‐voxel information is challenging because of the high dimensionality of the distributions.

The challenge of visualising the intricate and comprehensive information within diffusion MRI datasets is an active area of research (Leemans, 2010; Schultz & Vilanova, 2019) and very well established visualisation strategies exist to either convey the tensorial properties of a single voxel‐averaged **D** (Kindlmann, 2004; Pajevic & Pierpaoli, 2000; Westin et al., 1999) or to visualise a continuous ODF (Peeters, Prckovska, Almsick, Vilanova, & Romeny, 2009; Schultz & Kindlmann, 2010; Tournier et al., 2004; Tuch et al., 2002). However, such techniques are not immediately applicable to the discrete multi‐component distributions retrieved with our 5D correlation framework. Previously, we converted the retrieved distributions to sets of statistical parameter maps derived from either the entirety or sub‐divisions (bins) of the distribution space (de Almeida Martins et al., 2020; Topgaard, 2019). In de Almeida Martins et al. (2020), the *R*_2_ ‐ **D** space was divided into three bins capturing different ranges of **D** eigenvalues in order to separate the signal contributions from microscopic tissue environments with distinct diffusion properties. Even though bin‐resolved maps of signal fractions and means were observed to be useful to map sub‐voxel heterogeneity throughout the imaged brain volume, they do not provide information on orientation‐resolved properties. In this contribution, we demonstrate how *R*_2_ ‐ **D** distributions can be used to derive and visualise fibre‐specific relaxation and diffusion metrics. This is done by extending the binning procedure to the space of **D** orientations, and mapping discrete *P*(*R*_2_, **D**) components to a spherical mesh representing a dense set of orientation bins. The orientation‐resolved information is then conveyed as ODF glyphs that are colour‐coded according to the underlying relaxation and diffusion properties; this greatly facilitates the inspection and interpretation of the orientational variation of the 5D *P*(*R*_2_, **D**). The ODFs computed from the discrete ditributions are furthermore compatible with tractography algorithms which hence allows the extension to visualisation of longer‐range properties in 3D (Tax et al., 2015).

## 2 METHODS

### 2.1 Estimation of 5D relaxation–diffusion distributions

In diffusion MRI, heterogeneous tissues can be described as a collection of microscopic tissue environments wherein water diffusion is modelled by a local apparent diffusion tensor **D**. Within this multi‐tensor approach, the diffusion MRI signal is approximated as a weighted sum of the signals from the individual microscopic tissue environments (Jian, Vemuri, Özarslan, Carney, & Mareci, 2007; Novikov et al., 2019; Westin et al., 2016). A similar description has also been used in *R*_2_ studies of intra‐voxel brain tissue structure (Does, 2018; MacKay et al., 2006). The transverse relaxation signal of water within tissues is typically expressed as a multi‐exponential decay, given by the Laplace transform of a probability distribution of *R*_2_ values (Kroeker & Mark Henkelman, 1986; Whittall et al., 1997; Whittall & MacKay, 1989). Each coordinate of the relaxation probability distribution characterises the signal fraction of the microscopic environment with the corresponding *R*_2_ rate. Combining the relaxation and diffusion descriptions, the detected signal *S*(*τ*_E_,  **b**) can be written as

(1) $$S\left(\tau_{E},b\right)=S_{0}\int_{0}^{+\infty }\int_{{\mathrm{Sym}}^{+}\left(3\right)}P\left(R_{2},D\right)\;K\left(\tau_{E},b,R_{2},D\right)\;dD\;dR_{2},$$

where *P*(*R*_2_, **D**) is the continuous joint probability distribution of *R*_2_ and **D**, *τ*_E_ denotes the echo‐time, **b** is the diffusion‐encoding tensor, and *S*_0_ is the signal amplitude at vanishing relaxation‐ and diffusion‐weighting, that is, *S*_0_ = *S*(*τ*_E_ = 0, **b** = 0). The integration of **D** spans over the space Sym^+^(3) of symmetric positive semi‐definite 3 × 3 tensors. The kernel *K*(*τ*_E_, **b**, *R*_2_, **D**) encapsulates the signal decays mapping the distribution onto the detected signal. Here, we assume that the diffusion processes within each microscopic environment can be captured by an effective *R*_2_ and an apparent **D** that is related to a Gaussian distribution of gradient‐induced phase shifts, which in turns yields an exponentially decaying kernel: *K*(*τ*_E_, **b**, *R*_2_, **D**) = exp(−*τ*_E_*R*_2_)exp(−**b** : **D**) (Dell'Acqua et al., 2007; Does, 2018; Jian et al., 2007; Kaden et al., 2016; MacKay et al., 2006; Novikov et al., 2018; Scherrer et al., 2016; Tuch et al., 2002; Veraart, Novikov, & Fieremans, 2018; Westin et al., 2016).

Constraining the integral in Equation (1) to the space of axisymmetric diffusion tensors, each **D** can be parameterized by its axial and radial diffusivities, *D*_‖_ and *D*_⊥_, and by the polar and azimuthal angles, *θ* and *ϕ*, that define its orientation. The *D*_‖_ and *D*_⊥_ eigenvalues can in turn be combined to define measures of isotropic diffusivity *D*_iso_ = (*D*_‖_ + 2*D*_⊥_)/3 and normalised diffusion anisotropy *D*_Δ_ = (*D*_‖_ − *D*_⊥_)/(3*D*_iso_) (Eriksson, Lasič, Nilsson, Westin, & Topgaard, 2015). Using the popular approach of approximating the signal as a multi‐exponential decay (Dell'Acqua et al., 2007; Does, 2018; Jian et al., 2007; Kaden et al., 2016; MacKay et al., 2006; Novikov et al., 2018; Scherrer et al., 2016; Tuch et al., 2002; Veraart et al., 2018; Westin et al., 2016), considering only axisymmetric **b**, and adopting the (*D*_iso_, *D*_Δ_, *θ*, *ϕ*) parametrization, Equation (1) can be expanded as (de Almeida Martins & Topgaard, 2018).

(2) $$\frac{S\left(\tau_{E},b\right)}{S_{0}}=\int_{0}^{+\infty }\int_{0}^{+\infty }\int_{-1/2}^{1}\int_{0}^{\pi }\int_{0}^{2\pi }P\left(R_{2},D_{\mathrm{iso}},D_{\Delta },\theta ,\phi \right)K\left(...\right)d\phi \sin\theta d\theta dD_{\Delta }dD_{\mathrm{iso}}dR_{2},$$

with.

(3) $$K\left(...\right)=\exp\left(-\tau_{E}R_{2}\right)\exp\left(-{\mathrm{bD}}_{\mathrm{iso}}\left[1+2b_{\Delta }D_{\Delta }P_{2}\left(\cos\beta \right)\right]\right),$$

where *b* = Tr(**b**) is recognised as the traditional (scalar) *b*‐value and *b*_Δ_ denotes the normalised anisotropy of the diffusion‐encoding tensor (Eriksson et al., 2015). *P*_2_(*x*) = (3*x*^2^ − 1)/2 is the second Legendre polynomial, and *β* is the smallest angle between the symmetry axes of **D** and **b**. Note that each diffusion orientation (*θ*, *ϕ*) is associated with its own set of microscopic properties (*R*_2_, *D*_iso_, *D*_Δ_) and that no overarching microstructural kernel or universal orientation structure is assumed. This means that Equation (2) allows for fibre populations with distinct *R*_2_‐**D** properties.

For numerical implementation, Equation (2) is discretized as ***s*** = **K*w***, where ***s*** is the column vector of signal amplitudes measured with *M* combinations of (*τ*_E_, **b**) values, **K** is the inversion kernel matrix, and ***w*** is a vector containing the weights *w*_*n*_ of *N* discrete (*R*_2,*n*_,*D*_‖, *n*_,*D*_⊥, *n*_,*θ*_*n*_,*ϕ*_*n*_) configurations. The estimation of ***w*** can then be cast as a constrained linear least‐squares problem.

(4) $$w=\underset{w\geq 0}{\arg\min}{\left\|s-Kw\right\|}_{2}^{2}$$

In practice, the argument‐minimum operator in Equation (4) is replaced by a softer condition that searches for a solution within the noise variance. While seemingly straightforward, the problem of finding a solution whose residuals are compatible with the experimental noise is poorly conditioned. Indeed, multiple distinct solutions can be found to fit a single noisy dataset. This has motivated the development of several regularisation strategies in order to improve the stability of the inverse problem (Daducci et al., 2015; Mitchell, Chandrasekera, & Gladden, 2012; Provencher, 1982; Whittall & MacKay, 1989). A common strategy is to incorporate a regularisation term that promotes either a smooth (Benjamini & Basser, 2017; Provencher, 1982; Slator et al., 2019; Venkataramanan, Song, & Hurlimann, 2002) or a sparse (Benjamini & Basser, 2016; Berman, Levi, Parmet, Saunders, & Wiesman, 2013; Tax, Rudrapatna, Witzel, & Jones, 2017; Urbańczyk, Bernin, Koźmiński, & Kazimierczuk, 2013) ***w*** solution at the expense of a higher residual error.

Monte Carlo algorithms have been used in the porous media field as an alternative to conventional regularised approaches (de Almeida Martins & Topgaard, 2016, 2018; de Kort, van Duynhoven, Hoeben, Janssen, & Van As, 2014; Prange & Song, 2009). These algorithms purposely explore the variability between solutions and estimate an ensemble of distributions consistent with the experimental data. In this work, we use an unconstrained Monte‐Carlo algorithm specially designed to handle high‐dimensional correlation datasets (de Almeida Martins & Topgaard, 2018; Topgaard, 2019). The algorithm can be broadly divided in two iteration cycles. In the first cycle, the proliferation cycle, a user‐defined *N*_in_ number of points is randomly selected from the (log(*R*_2_), log(*D*_‖_), log(*D*_⊥_), cos*θ*, *ϕ*) space, and the corresponding set of weights is found by solving Equation (4) via a non‐negative linear least‐squares algorithm (Lawson & Hanson, 1974); points with non‐zero weights are stored and merged with a newly generated random set. This procedure is repeated for a total of *N*_p_ times, and *N*_p_ random sets of (*R*_2,*n*_,*D*_‖,*n*_,*D*_⊥,*n*_,*θ*_*n*_,*ϕ*_*n*_) components are sampled in order to find a configuration yielding sufficiently low residuals. The resulting {(*R*_2,*n*_,*D*_‖,*n*_,*D*_⊥,*n*_,*θ*_*n*_,*ϕ*_*n*_)} configuration is stored, duplicated, and its duplicate is then subjected to a small random perturbation. This initiates the second iteration cycle, named the mutation cycle, wherein configurations compete with their perturbed counterparts on the basis of lowest sum of squared residuals. The mutation cycle comprises a number of *N*_m_ rounds, following which a possible solution is estimated by selecting the points with the *N* highest weights. In this work we sampled *N*_in_ = 200 points from the (0 < log(*R*_2_/s^−1^) < 1.5,  −11.3 < log(*D*_‖_/m^2^s^−1^) <  −8.3,  −11.3 < log(*D*_⊥_/m^2^s^−1^) <  −8.3, 0 < cos*θ* < 1, 0 < *ϕ* < 2*π*) space, and used *N*_p_ = 20, *N*_m_ = 20, and *N* = 20. This inversion was performed voxel‐wise and bootstrap with replacement was used in order to estimate per‐voxel ensembles of *N*_b_ = 96 solutions, each of which consisting of 20 (*R*_2,*n*_,*D*_‖,*n*_,*D*_⊥,*n*_,*θ*_*n*_,*ϕ*_*n*_) components, {(*R*_2,*n*_,*D*_‖,*n*_,*D*_⊥,*n*_,*θ*_*n*_,*ϕ*_*n*_)}_1 ≤ *n* ≤ *N* = 20_, and their respective *w*_*n*_ weights.

### 2.2 Resolution of sub‐voxel fibre components

Spatially resolved 5D *R*_2_‐**D** distributions were estimated using the procedure described in the previous section. As the main brain components—white matter (WM), grey matter (GM), and cerebrospinal fluid (CSF)—are characterised by clearly distinct diffusion properties, we expect most (*R*_2,*n*_,*D*_‖,*n*_,*D*_⊥,*n*_,*θ*_*n*_,*ϕ*_*n*_) components to agglomerate within three distant regions of the diffusion space (Pierpaoli, Jezzard, Basser, Barnett, & Di Chiro, 1996).

The idea that most *P*(*R*_2_,**D**) components will fall within three coarse regions has inspired the division of the *R*_2_‐**D** space into three smaller subsets (bins) based on the diffusion properties of WM, GM, and CSF (de Almeida Martins et al., 2020). We then defined three bins named ‘thin’ (0.6 < log(*D*_‖_/*D*_⊥_) < 3.5, −10 < log(*D*_iso_/m^2^s^−1^) <  −8.7,   −0.5 < log(*R*_2_/s^−1^) < 2), ‘thick’ (−3.5 < log(*D*_‖_/*D*_⊥_) < 0.6,  −10 < log(*D*_iso_/m^2^s^−1^) <  −8.7, −0.5 < log(*R*_2_/s^−1^) < 2), and ‘big’ (−3.5 < log(*D*_‖_/*D*_⊥_) < 3.5, −8.7 < log(*D*_iso_/m^2^s^−1^) <  −8, −0.5 < log(*R*_2_/s^−1^) < 2). The names of the different bins highlight the geometry of the **D** captured by each one of them. For each bootstrap realisation *n*_b_ (1 ≤ *n*_b_ ≤ *N*_b_), signal contributions from anisotropic tissues are resolved by selecting the set of *P*(*R*_2_,**D**) components that fall within the ‘thin’ bin:

(5) $$\varepsilon_{n_{b}}^{\text{thin}}={\left\{\left(R_{2,i},D_{\|,i},D_{⊥,i},\theta_{i},\phi_{i},w_{i}\right)\right\}}_{n_{b},\;i\in \left\{\text{thin}\;\mathrm{bin}\right\}}.$$

**FIGURE 1:**
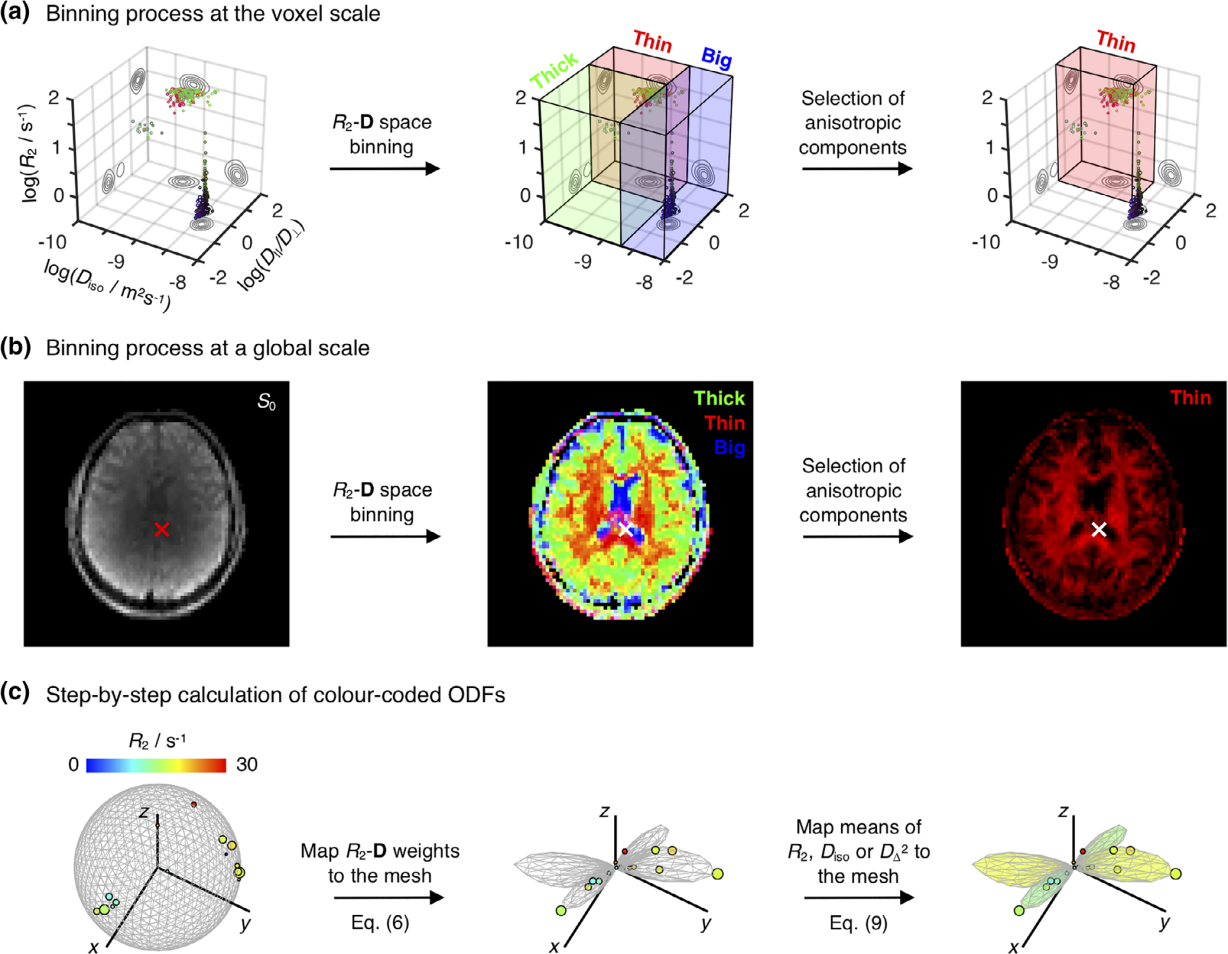
Resolution of sub‐voxel fibre‐like components and subsequent estimation of the associated colour‐coded Orientation Distributions Functions (ODFs). (a) *R*_2_‐**D** distribution obtained for a voxel containing both CSF and two crossing WM populations. The 5D *P*(*R*_2_,**D**) is reported as a 3D logarithmic scatter plot of *R*_2_, isotropic diffusivities *D*_iso_, and axial–radial diffusivity ratios *D*_‖_/*D*_⊥_, with circle area proportional to the weight of the corresponding *R*_2_‐**D** component, *w*. Colour coding is defined as: [R,G,B] = [cos*φ* sin*θ*, sin*ϕ* sin*θ*, cos*θ*] · |*D*_‖_−*D*_⊥_/max(*D*,*D*_⊥_), where (*θ*,*ϕ*) gives the orientation of each axisymmetric **D**. The *R*_2_‐**D** space is divided into three coarse bins named ‘big’ (blue volume), ‘thin’ (red volume), and ‘thick’ (green volume). Components falling in the ‘thin’ bin are singled‐out and interpreted as fibres. (b) Spatial distribution of per‐bin signal contributions. The middle map shows the fractional populations in the ‘big’ (blue), ‘thin’ (red), and ‘thick’ (green) bins as a colour‐coded composite image. The rightmost map focuses on the signal contributions from components within the ‘thin’ subset, *f*_thin_, the complement of which, (1 − *f*_thin_), gives the signal fraction from all components not used for ODF calculation. The crosses locate the voxel whose distribution is shown in panel (a). (c) Scheme for calculating colour‐coded ODFs. The *R*_2_‐coloured circles denote the ‘thin’ components from a bootstrap solution of the voxel signalled in panel (b). Circle area is proportional to *w*, while the [*x*,*y*,*z*] circle coordinates are defined as either [cos*ϕ* sin*θ*, sin*ϕ* sin*θ*, cos*θ*] (left) or [cos*ϕ* sin*θ*, sin*ϕ* sin*θ*, cos*ϕ*] · *w* (middle and right). In the left plot, the discrete *R*_2_‐**D** components are displayed on a unit sphere represented by a 1,000‐point (*θ*,*ϕ*) mesh. The weights of the *P*(*R*_2_,**D**) components are first mapped to the mesh through Equation (6), creating an ODF glyph whose radii inform on the *R*_2_‐**D** probability density along a given (*θ*,*ϕ*) direction (middle). Following the ODF estimation, Equation (9) is used to assign mean values of *R*_2_,*D*_iso_, or *D*_Δ_ to each mesh point and define the colour the ODF glyph (right)

The ${\left\{\left(R_{2,i},D_{\|,i},D_{⊥,i},\theta_{i},\phi_{i}\right)\right\}}_{n_{b},\;i\;\in \left\{\text{thin}\;\mathrm{bin}\right\}}$ configurations and ${\left\{w_{i}\right\}}_{n_{b},i\;\in }{}_{\left\{\text{thin}\;\mathrm{bin}\right\}}$ weights of $\varepsilon_{n_{b}}^{\text{thin}}$ are interpreted as representing the *R*_2_‐**D** properties and signal fractions, respectively, of a discrete set of sub‐voxel fibre populations. The binning and anisotropic selection processes are illustrated in panels A and B of Figure 1.

### 2.3 ODF estimation

The colour‐coded 3D scatter plots of *R*_2_, *D*_iso_ and *D*_‖_/*D*_⊥_ displayed in Figure 1 allow the visualisation of the full set of properties of the voxel‐wise $\varepsilon_{n_{b}}^{\text{thin}}$ components. Despite its usefulness, the scatter plot representation concentrates points corresponding to anisotropic components within a small region of the (*R*_2_,*D*_iso_,*D*_‖_/*D*_⊥_) space, which in turn makes it difficult to evaluate their orientation properties in detail. For example, while Figure 1a clearly informs on the existence of two fibre populations oriented along two different directions (red and green points), it does not provide unambiguous information about the relative signal contributions of the two populations. To better understand the orientational information of the underlying *P*(*R*_2_,**D**), it is helpful to convert the discrete set of fibre orientations to a continuous object informing on the *R*_2_‐**D** probability density in each direction, which can then be visualised as a single glyph with an intuitive geometrical interpretation. To this end, we used a *N*_verts_‐point triangle mesh on the unit sphere, created via an electrostatic repulsion scheme (Bak & Nielsen, 1997; Jones, Horsfield, & Simmons, 1999), to create *N*_verts_ uniformly distributed orientation bins; the mesh vertices define the centres of (*θ*, *ϕ*) bins. In this work, we used either *N*_verts_ = 3994 or *N*_verts_ = 1000 to define meshes where the median angular distance between nearest‐neighbouring points (or bin centres) is approximately 3.5° and 7°, respectively. Afterwards, a smoothing kernel was used to map the weights of $\varepsilon_{n_{b}}^{\text{thin}}$ onto the dense set of (*θ*,*ϕ*) bins. The role of the smoothing kernel is to weight the influence of each bin according to the angular distance between its centre and a given $\varepsilon_{n_{b}}^{\text{thin}}$ component, and to distribute the contributions of each discrete ${\left\{w_{i}\right\}}_{n_{b},i\in \left\{\text{thin}\;\mathrm{bin}\right\}}$ throughout various bins in order to define a smooth Orientation Distribution Function (ODF) $P_{n_{b}}\left(\theta ,\phi \right)$ that can be straightforwardly visualised as a polar plot (Leemans, 2010; Schultz & Vilanova, 2019). Alternatively, the process of mapping the discrete fibre components to a smooth ODF object can be cast as a kernel density estimation (KDE) exercise, where a smoothing kernel function with an appropriate bandwidth is used to estimate a continuous probability from a discrete dataset (Silverman, 1986).

In this work, the orientation binning and consequent estimation of $P_{n_{b}}\left(\theta ,\phi \right)$ was performed through a convolution with a Watson (Mardia & Jupp, 2009; Watson, 1965), or ‘spherical Gaussian’ (Harbison, Vogt, & Spiess, 1987), distribution kernel:

(6) $$P_{n_{b}}\left(\theta ,\phi \right)=\sum_{i\in \varepsilon_{n_{b}}^{\text{thin}}}w_{i}\exp\left[\kappa {\left(\mu \left(\theta ,\phi \right)\cdot u_{i}\right)}^{2}\right],$$

where ***u***_*i*_ is the unit vector describing the orientation of the *i*th discrete component and ***μ***(*θ*,*ϕ*) is the unit mean direction vector of bin centre (*θ*,*ϕ*). The variable *κ* denotes a concentration parameter that regulates the amount of dispersion about ***μ***(*θ*,*ϕ*). In the KDE parlance, *κ* is the bandwidth of the Watson smoothing kernel. Further insight into the nature of the Watson distribution kernel and the role of parameter *κ* is attained by considering the small‐angle approximation of the former.

(7) $$\exp\;\left[\kappa \cos^{2}\beta \right]\underset{\beta \to 0}{=}\exp\;\left[\kappa \right]\exp\;\left[-\kappa \beta^{2}\right]\;,$$

where *β* is the smallest angle between unit vectors ***u*** and ***μ***. Within this approximation, the Watson kernel is rewritten as a familiar Gaussian smoothing kernel whose standard deviation, *σ*, defines an angular spreading that is directly related to the concentration parameter *κ*, *σ* = (2*κ*)^−1/2^. The relationship between *κ* and *σ* enables us to easily set a dispersion factor in relation to the angular distance of the various mesh points. We set *σ* = 10.5° or, equivalently, *κ* = 14.9, that is, a *σ* parameter that is 50% larger than the median angular distance between nearest‐neighbouring vertices in a 1,000‐point triangle mesh. The rationale behind our choice of *κ* is elaborated upon in Section 3.1.

Separate ODFs were calculated for each of the *N*_b_ = 96 bootstrapped *P*(*R*_2_ **D**) solutions; the final *P*(*θ*,*ϕ*) was then estimated as the median of the *N*_b_ independent ODFs:

(8) $$P\left(\theta ,\phi \right)=\underset{n_{b}}{\mathrm{Med}}\left[P_{n_{b}}\left(\theta ,\phi \right)\right].$$

As the orientation of each fibre solution is correlated to a given set of {*R*_2,*n*_,*D*_‖,*n*_,*D*_⊥,*n*_} coordinates, we can assign any statistical descriptor of the (*R*_2_,*D*_iso_,*D*_Δ_) space to the various coordinates of *P*(*θ*,*ϕ*). In line with previous works (de Almeida Martins et al., 2020; Topgaard, 2019) where *R*_2_‐**D** were converted into maps of bin‐resolved mean *R*_2_, *D*_iso_, and $D_{\Delta }^{\;2}$, we map mean values of *R*_2_, *D*_iso_, and $D_{\Delta }^{\;2}$ into the ODF mesh. The mean value of *X* = *T*_2_, *R*_2_, *D*_iso_, or $D_{\Delta }^{\;2}$ per mesh orientation for each bootstrap *n*_b_, ${\hat{E}}_{n_{b}}\left[X\right]\left(\theta ,\phi \right)$, is calculated as

(9) $${\hat{E}}_{n_{b}}\left[X\right]\left(\theta ,\phi \right)=\frac{1}{P_{n_{b}}\left(\theta ,\phi \right)}\sum_{i\in \varepsilon_{n_{b}}^{\text{thin}}}w_{i}X_{i}\exp\left[\kappa {\left(\mu \left(\theta ,\phi \right)\cdot u_{i}\right)}^{2}\right].$$

By mapping different descriptors of the *R*_2_‐**D** distributions to specific ODF coordinates, we can visualise orientation‐specific information on tissue composition and structure. As before, the final voxel‐wise $\hat{E}\left[X\right]\left(\theta ,\phi \right)$ is estimated as the median of the individual per‐bootstrap ${\hat{E}}_{n_{b}}\left[X\right]\left(\theta ,\phi \right)$ values:

(10) $$\hat{E}\left[X\right]\left(\theta ,\phi \right)=\underset{n_{b}}{\mathrm{Med}}\left[{\hat{E}}_{n_{b}}\left[X\right]\left(\theta ,\phi \right)\right]\equiv \hat{E}\left[X\right].$$

For compactness, we omit the explicit (*θ*,*ϕ*) dependence from the orientation‐resolved means and simply denote them as $\hat{E}\left[X\right]$. The $\hat{\text{E}}\left[D_{\text{iso}}\right]$ metric provides orientation‐resolved information on the underlying mean‐diffusivity. The $\hat{\text{E}}\left[D_{\Delta }^{2}\right]$ metric is the orientation‐resolved counterpart of the mean $D_{\Delta }^{2}$ descriptor (Topgaard, 2019), which is in turn similar to previously introduced anisotropy measures such as the microscopic anisotropy index (MA; Lawrenz, Koch, & Finsterbusch, 2010), the fractional eccentricity (FE; Jespersen, Lundell, Sønderby, & Dyrby, 2013), and the microscopic fractional anisotropy (μFA; Lasič, Szczepankiewicz, Eriksson, Nilsson, & Topgaard, 2014).

The local maxima of fibre ODFs, commonly referred to as ‘peaks’, have been used to quantify the number of per‐voxel fibres and their respective orientations (Dell'Acqua, Simmons, Williams, & Catani, 2013; Jeurissen et al., 2014; Jeurissen, Leemans, Tournier, Jones, & Sijbers, 2013). Here, we follow this traditional procedure and extend it to assign *R*_2_‐**D** metrics to the (*θ*,*ϕ*) coordinates of each ODF peak. Up to four peaks per voxel were determined by assessing the mesh points (*θ*,*ϕ*) for which *P*(*θ*,*ϕ*) is a local maximum and *P*(*θ*,*ϕ*)/max_(θ,ϕ)_[*P*(*θ*,*ϕ*)] ≥ 0.1. The *R*_2_‐**D** properties of the ODF peaks were estimated by calculating $\hat{E}\left[X\right]$ (see Equation (9)) for each peak orientation. The performance of the peak‐based metrics is dependent on the size of the mesh used to construct the smooth ODF objects, with a higher number of mesh points resulting in lower biases irrespective of the set value of *κ*. With higher mesh sizes leading to significantly longer computational times, we have opted to use a 3,994‐point mesh for peak calculations as a good compromise between accuracy and computational effort.

The mapping of *P*(*R*_2_,**D**) components to a dense mesh as described by Equations (6)–(10) is a key result from this contribution, and provides the basis for extracting and visualising orientation‐resolved information from nonparametric *R*_2_‐**D** distributions. Figure 1c illustrates how both $\hat{\text{E}}\left[X\right]$ and the associated orientation distribution can be conveniently represented by colour‐coded ODF glyphs; the shape of the glyph reflects the *P*(*θ*,*ϕ*) distribution, while the colour informs on the $\hat{\text{E}}\left[X\right]$ values at the various (*θ*,*ϕ*) points. Functions used to compute the colour‐coded ODFs and their associated peaks have been incorporated in the multidimensional diffusion MRI toolbox (Nilsson et al., 2018): https://github.com/JoaoPdAMartins/md-dmri. In this work, maps of ODF glyphs were computed using those same functions on a 1,000‐point mesh and rendered with POV‐Ray (http://www.povray.org/).

### 2.4 Real‐time multi‐peak tractography

Streamlines were generated and visualised in real‐time using FiberNavigator (Chamberland, Whittingstall, Fortin, Mathieu, & Descoteaux, 2014). Tracking was performed on the extracted ODF peaks using 8 seeds per voxel and the following parameters: step size = 1 mm, maximum angle = 50°, minimum length = 10 mm, and maximum length = 200 mm.

### 2.5 In vivo data acquisition

Multidimensional relaxation–diffusion MRI data were acquired using a prototype spin‐echo diffusion weighted sequence with an echo‐planar imaging (EPI) readout, customised for diffusion encoding with user‐defined gradient waveforms (Lasič et al., 2014; Szczepankiewicz, Sjölund, Stahlberg, Latt, & Nilsson, 2019). Images were recorded with the following parameters: TR = 4 s, FOV = 234 × 234 × 60 mm^3^, voxel ‐ size = 3 × 3 × 3 mm^3^, partial ‐ Fourier = 6/8, and a parallel‐imaging (GRAPPA) factor of 2. Diffusion encoding was performed with a set of five gradient waveforms yielding *b*‐tensors with four distinct ‘shapes’ (*b*_Δ_ =  − 0.5, 0.0, 0.5, and 1) (Eriksson et al., 2015). The various waveforms were used to form *b*‐tensors of varying magnitude *b*, anisotropy *b*_Δ_, and orientation (*θ*,*ϕ*) at different echo‐times *τ*_E_; this procedure yields 5D relaxation–diffusion correlated datasets whose dimensions match those of the sought‐for distributions. Readers interested in the sequence used in this work are directed to a public repository: https://github.com/filip-szczepankiewicz/fwf_seq_resources.

Table 1 summarises the (*τ*_E_,**b**) acquisition protocol. Besides the (*τ*_E_,**b**) points detailed in Table 1, we additionally acquired *b* = 0 images with reversed phase‐encoding blips at *τ*_E_ = 60, 80, 110, and 150 ms in order to correct for susceptibility‐induced distortions (Andersson, Skare, & Ashburner, 2003). The acquisition scheme comprised a total of 686 (*τ*_E_,**b**) acquired over an imaging session of ~45 min. The diffusion‐encoding waveforms used in this work are displayed in Figure S1 of the Supporting Information. Waveforms giving *b*_Δ_ =  − 0.5, 0.0, and 0.5 were calculated with a MATLAB package (https://github.com/jsjol/NOW) that optimises for maximum *b* (Sjölund et al., 2015) and minimises the effects of concomitant magnetic field gradients (Szczepankiewicz, Westin, & Nilsson, 2019). Linearly encoded (*b*_Δ_ = 1) data were acquired with two different gradient waveforms: a non‐monopolar gradient waveform and a standard Stejskal–Tanner waveform (Stejskal & Tanner, 1965). The asymmetric gradient pulses from the non‐monopolar waveform were designed with the aim of minimising the differences between the spectral profile of such *b*_Δ_ = 1 waveform and the spectral profile of the *b*_Δ_ ≠ 1 waveforms (Lundell et al., 2019). The Stejskal–Tanner design was used to probe a shorter *τ*_E_ and higher *b*‐values (*b* = 4 × 10^9^ m^−2^s) than those achievable with the non‐monopolar *b*_Δ_ = 1 waveform. While the measured apparent diffusivities are known to be related to the frequency spectra of the gradient waveforms (Callaghan & Stepišnik, 1996; Lundell et al., 2019; Stepišnik, 1993), such a relationship is likely to have a negligible effect on healthy human brain data acquired with the limited range of frequency contents probed in this work (Szczepankiewicz et al., 2019) and no biases are expected to originate from the spectral differences of the *b*_Δ_ = 1 waveforms.

**TABLE 1:** 5D relaxation–diffusion correlation protocol used in this work.

	*τ*_E_ (10^−3^ s)	*b*‐values (10^9^ m^−2^s)	#directions/*b*‐value	Number of points
*b*_Δ_ = 1, ST	60	0.0, 0.1, 0.7, 1.4, 2.0	6, 6, 12, 30^a^	54
*b*_Δ_ = 1, ST	80	0.0, 0.1, 0.8, 2.0, 4.0	6, 6, 16, 50^a^	78
*b*_Δ_ = 1	80, 110, 150	0.0, 0.1, 0.7, 1.4, 2.0	6, 6, 12, 30^a^ ^,^ ^b^	162
*b*_Δ_ = 0.5	80, 110, 150	0.0, 0.1, 0.7, 1.4, 2.0	6, 6, 10, 16^a^ ^,^ ^b^	114
*b*_Δ_ = 0	80	0.0, 0.3, 1.0, 2.0	6, 6, 6^c^	108
*b*_Δ_ = 0	80, 110, 150	0.0, 0.1, 0.7, 1.4	4, 4, 4^b^	36
*b*_Δ_ = −0.5	80, 110, 150	0.0, 0.1, 0.7, 1.4, 2.0	6, 6, 10, 16^a^ ^,^ ^b^	114

^a^ Directions generated using electrostatic repulsion on the half‐sphere (Bak & Nielsen, 1997; Jones et al., 1999).

^b^ Repeated for all *τ*_E_ values.

^c^ Repeated for six different permutations of the [*G*_*x*_,*G*_*y*_,*G*_*z*_] components of the *b*_Δ_ = 0 waveform.

The protocol described above was implemented on a 3 T Siemens MAGNETOM Prisma scanner (Siemens Healthcare, Erlangen, Germany) and used to scan a healthy adult volunteer. This study was approved by the Cardiff University School of Psychology ethics committee, and informed written consent was obtained prior to scanning.

### 2.6 Post processing

The entire dataset was divided in *τ*_E_‐specific data subsets, which were denoised using random matrix theory (Veraart et al., 2016), and corrected for Gibbs ringing artefacts using the method described in (Kellner, Dhital, Kiselev, & Reisert, 2016). Signal drift correction was subsequently performed as detailed in Vos et al. (2017). The acquired data were further corrected for subject motion and eddy‐current artefacts using ElastiX (Klein, Staring, Murphy, Viergever, & Pluim, 2009) with extrapolated references (Nilsson, Szczepankiewicz, van Westen, & Hansson, 2015) as implemented in the multidimensional diffusion MRI toolbox (Nilsson et al., 2018); this procedure was performed with the default settings of the toolbox to the entire (*τ*_E_,**b**) dataset. Susceptibility‐induced geometrical distortions were corrected using the TOPUP tool in the FMRIB software library (FSL; Smith et al., 2004), with the same settings being applied to the entire (*τ*_E_,**b**) dataset.

### 2.7 In silico datasets

In silico data were used to investigate the angular resolution and the performance of the suggested acquisition and analysis protocols. We simulated a multi‐component system designed to mimic up‐to‐three crossing fibres with similar diffusion features (*D*_iso_ = 0.75 ⋅ 10^−9^m^2^s^−1^,  *D*_Δ_ = 0.9) but distinct orientations and relaxation properties:

- Component 1: *T*_2_ = 60 ms, *θ* = 0°, *ϕ* = 0°, *w* = *f*_thin_/*n*_fibre_;
- Component 2: *T*_2_ = *T*_2, cross_, *θ* = *θ*_cross_, *ϕ* = 0°, *w* = *f*_thin_/*n*_fibre_;
- Component 3: *T*_2_ = *T*_2, cross_, *θ* = *θ*_cross_, *ϕ* = 90°, $w=\left\{\begin{array}{rr} 0, & \text{if}\;n_{\text{fibre}}=2\\ f_{\text{thin}}/n_{\text{fibre}}, & \text{if}\;n_{\text{fibre}}=3 \end{array}\right)$.

To assess the angular resolution, we simulated a two‐fibre system with different fibre‐specific relaxation times by setting *f*_thin_ = 1, *n*_fibre_ = 2, and *T*_2,cross_ = 100 ms. The polar angle of component 2 was varied in order to define four distinct fibre crossing angles: *θ*_cross_ = 25°, *θ*_cross_ = 30°, *θ*_cross_ = 35°, and *θ*_cross_ = 40°.

A more comprehensive set of systems was simulated to thoroughly test the performance of the proposed framework. Two‐ and three‐fibre systems were designed by setting either *n*_fibre_ = 2 or *n*_fibre_ = 3, respectively. Different fibre‐crossings including inter‐fibre *T*_2_ differences were simulated by sampling unique combinations of (*T*_2,cross_,*θ*_cross_) parameters, where *T*_2,cross_ ∈ {60, 70, 80, 90, 100} ms and *θ*_cross_ ∈ {0°,  15°,  30°,  60°,  90°}. A fourth component mimicking the coarse *R*_2_‐**D** properties of GM (*T*_2_ = 90 ms, *D*_iso_ = 0.8 ⋅ 10^−9^ m^2^s^−1^, *D*_Δ_ = 0.2) was subsequently added at varying signal fractions, 1 − *f*_thin_ ∈ {0, 0.3, 0.5, 0.7, 0.9}.

The ground‐truth signal data for the various fibre‐crossing systems were generated using the (*τ*_E_,**b**) acquisition scheme indicated in Table 1 and computed from Equation (2). Gaussian distributed noise with an amplitude of 1/SNR was added to the ground‐truth signals in order to simulate the effects of experimental noise. The experimental SNR, computed as the mean‐to‐standard‐deviation‐ratio of the *b*_Δ_ = 0 data acquired at *b* = 0.3 ⋅ 10^9^ m^−2^s and *τ*_E_ = 80 ms (Szczepankiewicz, Sjölund, et al., 2019), was estimated to SNR = 72 ± 28 for WM regions. Consequently, we defined SNR = 70 for the in silico calculations, a value that is compatible with the SNR of the in vivo data. In line with a recent in silico study of the performance of the Monte Carlo algorithm in inverting multidimensional diffusion data (Reymbaut, Mezzani, de Almeida Martins, & Topgaard, 2020), we drew *N*_noise_ independent noise configurations and computed *N*_noise_ different signal realisations for each of the in silico systems; we used *N*_noise_ = 100 for evaluating the angular resolution of the framework and *N*_noise_ = 40 in simulations for performance testing. The various signal realisations were inverted using the Monte‐Carlo algorithm described in Section 2.1, and the resulting solution ensembles were subsequently compared against the corresponding ground‐truth systems.

## 3 RESULTS AND DISCUSSION

### 3.1 Defining the dispersion factor of the Watson kernel

As mentioned in Section 2.3, the use of a Watson kernel introduces an artificial angular dispersion to the inverted $\varepsilon_{n_{b}}^{\text{thin}}$ components. The amount of angular dispersion is regulated by the user‐defined parameter *κ*, which should be large enough not to over‐smooth the data and sufficiently small to avoid spurious peaks within an individual sample.

To understand the smoothing effects of the Watson kernel over a discrete mesh, it is instructive to consider the decay of the Watson function over a given angular distance *Δβ* : *ν* = (exp[*κ*cos^2^*Δβ*] − 1)/(exp[*κ*] − 1). Considering a 1,000‐point mesh and *κ* = 14.9, values used for the in vivo data visualisation, the maximum distance between an arbitrary ${\left\{\theta_{i},\phi_{i}\right\}}_{n_{b},\;i\in \left\{\text{thin}\;\mathrm{bin}\right\}}$ configuration and the nearest mesh point is ~3.5°, a value for which the Watson kernel retains *ν* = 0.95 of its maximal influence. The minimal decay of the Watson kernel over *Δβ* = 3.5° ensures that the set of $\varepsilon_{n_{b}}^{\text{thin}}$ discrete components is indeed mapped into the mesh. From Equation (7) it is additionally obvious that the choice of *κ* is a trade‐off between a sufficiently smooth ODF representation and the angular resolution of the ODF in disentangling different peaks. The question then arises whether setting *κ* = 14.9 may over‐smooth the orientational information within the *R*_2_‐**D** distributions. For instance, with *κ* = 14.9 the Watson kernel will retain more than 50% (*ν* = 0.64) of its maximum value over a distance of *Δβ* = 10°, meaning that 20° crossings cannot be resolved with our settings. To assess if the amount of *κ*‐generated dispersion is sufficiently low not to misrepresent the orientational information of the *R*_2_‐**D** distributions, we investigated *in silico* the angular resolution of the Monte‐Carlo analysis.

**FIGURE 2:**
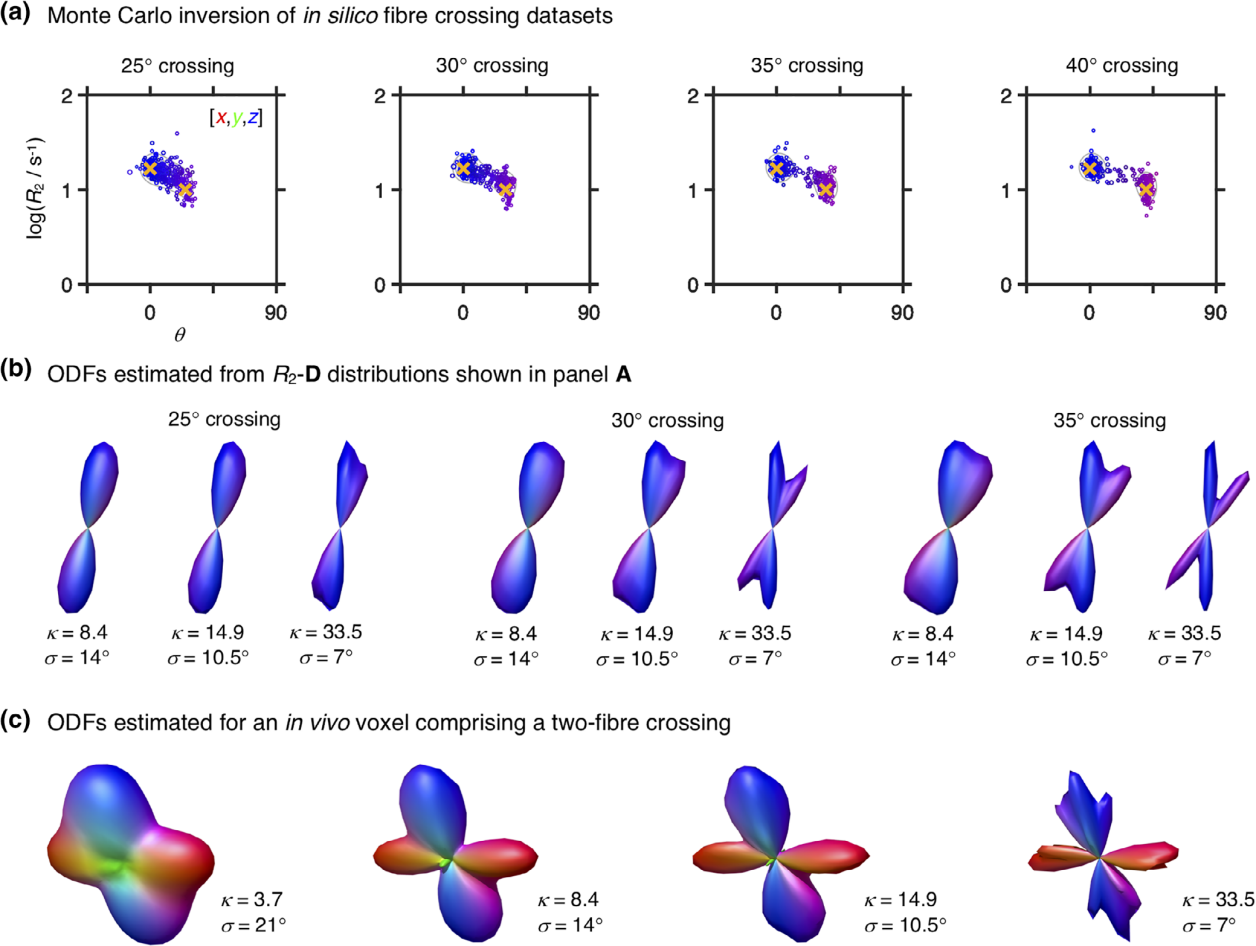
*R*_2_‐**D** distributions and Orientation Distribution Functions (ODF) retrieved for in silico fibre‐crossing datasets. (a) 5D *P*(*R*_2_,**D**) distributions displayed as 2D scatter plots of log(*R*_2_) and *θ*, the polar angle defining **D** orientation. Circle area is proportional to the weight of the corresponding component and colouring is defined as R,G,B] = [cos*ϕ* sin*θ*, sin*ϕ* sin*θ*, cos*θ*] · |*D*_‖_ − *D*_⊥_/max(*D*_‖_,*D*_⊥_), where *D*_‖_ and *D*_⊥_ denote the axial and radial diffusivities, respectively, and *ϕ* is the azimuth angle of **D**. The yellow crosses identify the ground‐truth values. (b) ODF glyphs estimated from the distributions in panel (a), using Watson kernel with different orientation dispersion factors (see Equation (6) for further details). The ODF colouring follows a conventional directional scheme: [R,G,B] = [*μ*_*xx*_,*μ*_*yy*_,*μ*_*zz*_], where *μ*_*ii*_ are the elements of the unit vector ***μ***(*θ*,*ϕ*) defining the orientation of mesh‐point (*θ*,*ϕ*). (c) ODF glyphs estimated for an in vivo voxel rendered as triangular surface plots for varying angular standard deviation *σ* of the convolution kernel at constant number of triangle vertices and the same underlying 5D *P*(*R*_2_,**D**) distribution. The depicted voxel comprises a two‐way crossing between fibres from the corticospinal tract and the *corpus callosum*

The angular resolution of our framework was assessed by inverting *in silico* data from two anisotropic components crossing at various angles (see Section 2.6 for further details). The (*R*_2_,*θ*) projections of the attained *R*_2_‐**D** distributions are displayed in Figure 2a and inform that, at the SNR of the in vivo data, crossings of 30° or higher can be directly resolved in the Monte Carlo *P*(*R*_2_,**D**). Setting equal *R*_2_ (*T*_2_ = 1/*R*_2_ = 60 ms) properties for both anisotropic did not affect the angular resolution of the 30° crossing, but lead to an overestimation of the signal fraction from the *θ* = 30° fibre population. The accurate resolution of the 35° and 40° systems was unaffected by changes in component *R*_2_. The in silico results then suggest that the maximum achievable angular resolution of our experimental protocol is between 30° and 35°, and a conservative approach is to set *κ* so that 35° crossings are not over‐smoothed and obscured. Computing ODFs for the in silico distributions confirms that setting *κ* = 14.9 is indeed sufficient to unambiguously resolve a 35° crossing (Figure 2b). While there is room to increase *κ* without risking $\varepsilon_{n_{b}}^{\text{thin}}$ disappearing though the holes of the mesh, we observe that a significantly sharper Watson kernel leads to narrow ODF lobes that do not accurately portray the angular dispersion of the underlying *R*_2_‐**D** distributions (compare panels **A** and **B** of Figure 2). Moreover, significantly higher *κ* values were tested in the in vivo dataset and observed to lead to non‐smooth ‘spiky’ ODFs in voxels containing orientationally dispersed fibres (see Figure 2c).

As depicted in Figure 2c, the choice of *κ* has a clear effect on the computed ODF, which should in turn be interpreted in light of that same choice. For instance, the width of the computed ODF lobes is a product of both the angular dispersion of the underlying fibre system and the choice of *κ*. Similarly, an upper limit to the achievable angular resolution is also implicitly set by the choice of *κ* parameter and the related angular standard deviation of the convolution kernel; by setting *κ* = 14.9 (*σ* = 10.5°) we render it impossible to resolve crossings of 20° or lower angles, independently of the angular resolution of the basis *R*_2_‐**D** distributions. In this work, the angular distance between near‐neighbouring mesh‐points and the angular resolution of the *P*(*R*_2_,**D**) solutions were used to define the upper and lower limits, respectively, of the *κ* parameter. An alternative promising approach would be to build upon prior literature on bandwidth optimisation in KDE algorithms (Cao, Cuevas, & González Manteiga, 1994; Park & Marron, 1990) in order to devise methods for automatic *κ* selection.

### 3.2 In silico ODF and peak metrics estimation

**FIGURE 3:**
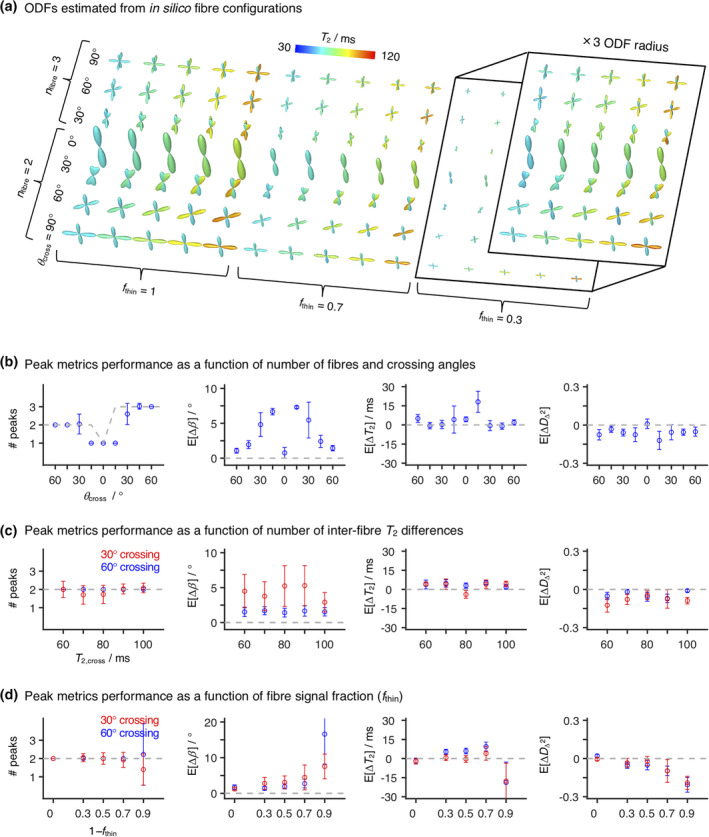
Orientation Distribution Functions (ODF) and peak metrics retrieved for in silico fibre‐crossing datasets. (a) ODF glyphs computed for the in silico systems described in Section 2.6. The displayed ODFs are coloured according to the orientation‐resolved means of *T*_2_, Ê[*T*_2_] (see Equation (9) for more details). The inset displays the *f*_thin_ = 0.3 ODFs with their base amplitude rescaled by a factor of three. (b–d) Number of peaks (# peaks) and mean biases (E[Δ*X*]) in peak angle (b), peak *T*_2_, and peak squared normalised diffusion anisotropy $D_{\Delta }^{2}$ estimated for three selected in silico systems. We refer the reader to Section 2.6 for a detailed description of the designed systems. Briefly, the displayed systems were designed by combining upto three discrete fibre components (*D*_iso_ = 0.75 × 10^–9^ m^2^ s^–1^, *D*_Δ_ = 0.9) with different signal fractions (1 − *f*_thin_) of a GM‐like component (*T*_2_ = 90 ms, *D*_iso_ = 0.8 × 10^–9^ m^2^ s^–1^, *D*_Δ_ = 0.2). Here, we inspect a set of *n*_fibre_ discrete fibres at various crossing angles *θ*_cross_ (b), two‐fibre crossings for various *T*_2_ values of the crossing fibre, *T*_2,cross_ (c), and two‐fibre crossings with varying *f*_thin_ (d). In (b) and (c), we set 1 − *f*_thin_ = 0.3, while in (b) and (d), we selected *T*_2,cross_ = 80 ms. The metrics were calculated across 40 different noise realisations; the points represent the mean over the various signal realisations, while the error bars indicate the standard deviation across signal realisations. The dashed grey line indicates the true number of peaks (left plots) or the zero‐bias line

Figure 3a shows the ODF glyphs recovered from the in silico datasets described in Section 2.6. For relatively high signal fractions of fibre components (*f*_thin_ = 1 or 0.7), the simulated orientational configurations are accurately captured and the inter‐fibre *T*_2_ differences detected, with the higher *T*_2_ fibres being correctly identified. Worse performance is found at lower fibre signal fraction *f*_thin_ and lower crossing angles *θ*_cross_ = 30°. In particular, we notice that, at *f*_thin_ = 0.3 and *θ*_cross_ = 30°, the proposed framework cannot accurately capture three‐fibre crossings and that it misestimates the ODF lobe amplitude of the (*θ* = 0°, *ϕ* = 0°) fibre for two‐fibre systems. Less noticeable biases in ODF lobe amplitudes can also be found in two‐ and three‐fibre crossings with *θ*_cross_ = 60°. While biases in relaxation time can be detected throughout the *f*_thin_ = 0.3 systems, we note that the higher *T*_2_ fibres are correctly identified and inter‐fibre *T*_2_ contrast is somewhat preserved.

Peak‐based metrics were derived from the in silico ODFs. To evaluate the accuracy and precision of the estimated peaks and their associated metrics, we computed the angular distance between each estimated peak and the various ground‐truth fibre components, and assigned each peak to its closest ground‐truth fibre. Following the peak assignment, we estimated the differences (biases) between the orientations and *R*_2_‐**D** properties of the estimated peaks and their corresponding ground‐truth components.

The peak metrics biases displayed in Figure 3b–d confirm some of the observations made in previous paragraphs. For example, significantly higher mean angular biases E[*Δβ*] are found for *θ*_cross_ = 30° crossings (see E[*Δβ*] plots in Figure 3b,c), thus confirming the observations of Section 3.1 which pointed to a maximum angular resolution of 30–35°. Consistently, we also observe a large uncertainty in the number of peaks detected in 30° crossing systems. We also observe that, with the current settings, only a single peak is detected for 15° crossings; this is consistent with the fact that a *κ* of 14.9 does not allow the angular separation of fibres crossing at less than 20°. For crossings at 45° or higher angles, the number of peaks is accurately and precisely recovered, and we register E[*Δβ*] of less than 2.5°. These biases are comparable to those found in ref. (Jeurissen et al., 2014), where similarly designed simulations were deployed to validate a suggested multi‐tissue spherical deconvolution approach. Moreover, as evidenced by Figure 3c,d, the angular resolution of the *θ*_cross_ = 60° system is unaffected by variations in *T*_2,cross_ or partial voluming with lower anisotropy components as long as the fibre components account for more than 30% of the signal at (*τ*_E_ = 0, **b** = 0). Focusing on the *T*_2_ and $D_{\Delta }^{2}$ peak metrics, we notice a slight mean positive bias in *T*_2_ and a mean negative bias in $D_{\Delta }^{2}$. Both biases seem to be independent of variations in *T*_2,cross_, but a progressive underestimation of the anisotropy of the fibre components with decreasing *f*_thin_ is observed (see rightmost plot of Figure 3d). While not displayed, mean biases of ±0.1 × 10^−9^ m^2^s^−1^ were estimated for the *D*_iso_ metric for systems with *f*_thin_ > 0.1, thus indicating a good performance of peak‐based metrics in assessing orientation‐resolved mean diffusivities.

### 3.3 In vivo fibre orientations

Previous work from our group (de Almeida Martins et al., 2020) has shown that voxels containing just one tissue type (WM, GM, or CSF) give rise to distinct *R*_2_‐**D** distributions that accurately capture the main microscopic features of the various tissues—CSF: high isotropic diffusivity *D*_iso_, low normalised diffusion anisotropy *D*_Δ_, low *R*_2_; WM: low *D*_iso_, high *D*_Δ_, high *R*_2_; GM: low *D*_iso_, low *D*_Δ_, high *R*_2_. Voxels comprising mixtures of WM, GM, and CSF are in turn characterised by multimodal distributions that exhibit a linear combination of properties of the distributions from the individual components. Figure 1a displays the distribution obtained from a voxel containing both CSF and contributions from two WM tracts: the *corpus callosum* (CC) and the *fornix*. Three distinct tissue environments can be clearly discerned: an isotropic fast diffusing component attributed to CSF and two anisotropic slow diffusing components with different orientations corresponding to the WM tracts. By ascribing distribution points to one of the three bins discussed in the Methods section, we were able to separate and quantify the signal contributions from distinct brain tissues. Indeed, as shown in Figure 1b, the signal fractions from the various bins follow the expected spatial distributions of WM, GM, and CSF.

**FIGURE 4:**
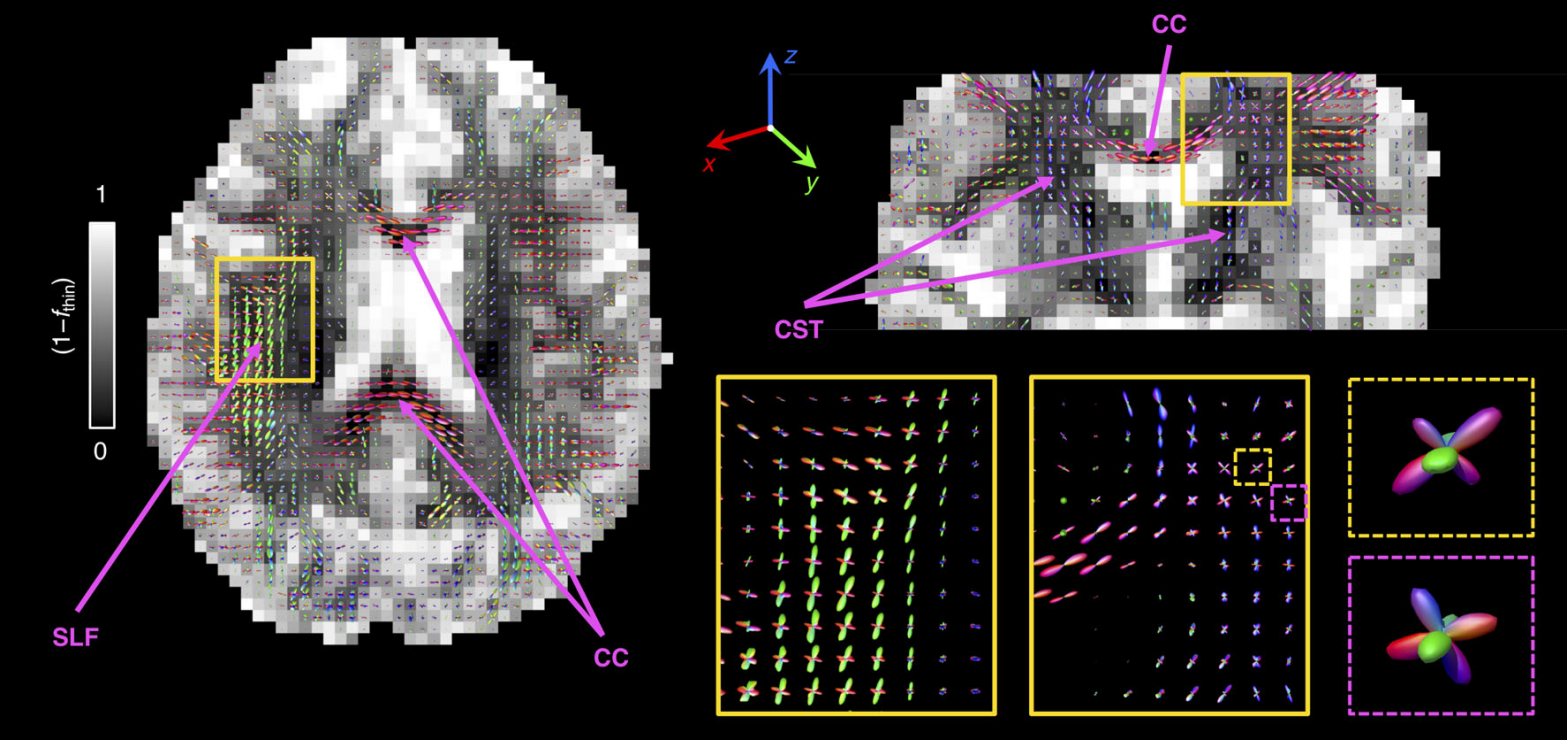
Per‐voxel Orientation Distribution Functions (ODF), *P*(*θ*,*ϕ*), estimated from *R*_2_‐**D** distribution components ascribed to the ‘thin’ bin defined in Figure 1. The voxel‐wise *P*(*θ*,*ϕ*) were computed by using Equation (6) to map the weights of the bin‐resolved discrete *P*(*R*_2_,**D**) components into a 1,000‐point spherical mesh. Here, each ODF is represented as a 3D polar plot with a local radius given by *P*(*θ*,*ϕ*) and colour‐coded according to [R,G,B] = [*μ*_*xx*_,*μ*_*yy*_,*μ*_*zz*_], where *μ*_*ii*_ are the elements of the unit vector ***μ***(*θ*,*ϕ*) (see Equation (6) for further details). In the left and top‐right panels, the sets of ODF glyphs are superimposed on a grey‐scaled map that shows the signal contributions from non‐fibre‐like components (1 − *f*_thin_), that is, signal fractions from the ‘big’ and ‘thick’ populations. The zoom‐ins in the lower‐right panel offer a more detailed look into selected fibre crossing regions (continuous line boxes) and three‐fibre crossing voxels (dashed line boxes) found in the *centrum semiovale*. The various arrows identify fibre tracts mentioned in the main text

Figure 4 displays the ODFs computed from the components that fall within the ‘thin’ bin. The ODFs are displayed as directionally coloured glyphs, superimposed on the sum of the signal fractions from the ‘big’ and ‘thick’ populations. Overall, the reconstructed ODFs are consistent with the expected WM arrangement of the healthy human brain. Major WM tracts such as the corticospinal tract (CST), the CC, and the superior longitudinal fasciculus (SLF) are easily located (see arrows in Figure 4), and multiple crossings can also be discerned. The zoomed panels show that the proposed method can capture the crossings in the ventral SLF—anterior–posterior fibres with left–right fibres—and the crossings between the CST and the CC*—*superior–inferior fibres with left–right fibres. The dotted boxes show that three‐fibre crossings present in the *centrum semiovale* are well captured by this technique, meaning that more than two fibre populations can be resolved.

Voxels at the WM‐CSF and WM‐GM interfaces exhibit small‐amplitude ODFs, consistent with lower signal fractions of fibrous tissue. The low amplitude of the ODF lobes found in those regions does not seem to bias their orientation; for example, CC voxels near the ventricles yield low amplitude lobes whose orientations follow the expected trend (fibres running left–right). These observations indicate that the estimated ODFs are robust to partial volume effects with CSF and that the proposed method can indeed resolve fibre orientations in heterogeneous voxels. In silico simulations show that an accurate ODF can be estimated as long as the contribution from CSF accounts for less than 75% of the total voxel‐signal at (*τ*_E_ = 0, **b** = 0). Low‐amplitude ODF lobes can also be found throughout cortical GM regions. These ODFs might be explained by the presence of anisotropic tissue components in cortical GM (Assaf, 2019), or interpreted as originating from low‐amplitude WM partial volume effects caused by the large voxel‐size used in this study. A more in‐depth study is necessary in order to unambiguously discriminate between these two factors.

**FIGURE 5:**
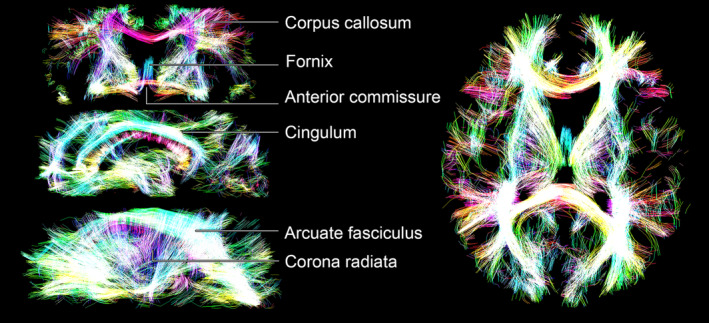
Opacity rendering of streamline tractography data, where the opacity reflects streamline density (computed using a slice thickness of 3 voxels). The various tracks are coloured according to their orientation: red (left‐right), green (anteroposterior), and blue (superoinferior). The right‐side panels display one coronal slice (top) and two different sagittal slices (middle and bottom), while the left‐side panel displays an axial slice

Figure 5 shows multi‐peak tractography based on the peak‐directions of the ODFs generated from the 5D *R*_2_‐**D** distributions. Multiple well‐known fibre bundles can be recognised and are annotated in the figure. The displayed tracks confirm that anatomically‐plausible WM pathways can indeed be extracted from the ODF maps of Figure 4.

### 3.4 In vivo orientation‐resolved ***R***_**2**_ ‐ **D** metrics

**FIGURE 6:**
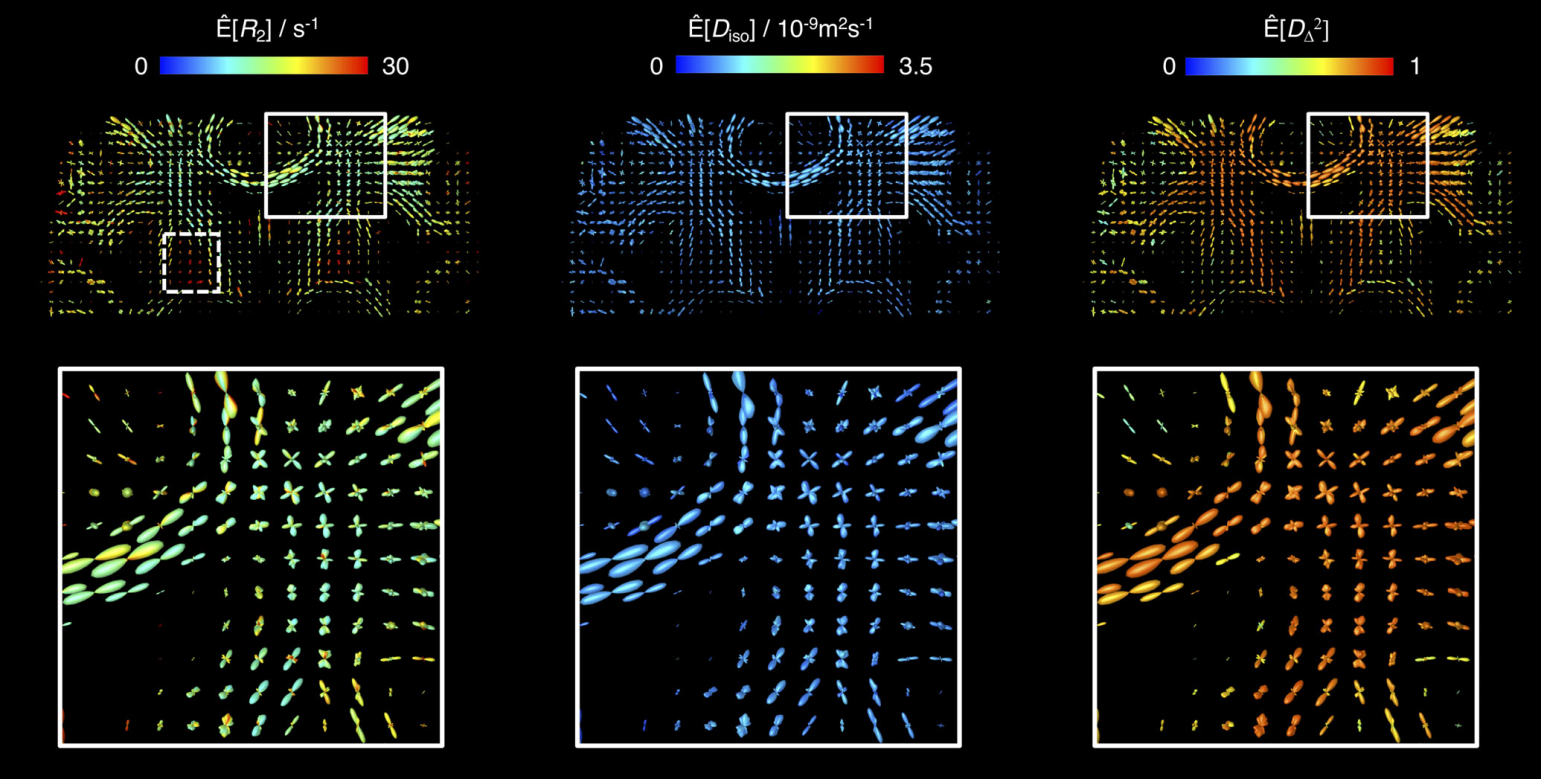
Orientation Distribution Function (ODF) maps coloured according to orientation‐resolved means, Ê[*X*], of *R*_2_, isotropic diffusivity *D*_iso_, and squared normalised diffusion anisotropy $D_{\Delta }^{2}$. All Ê[*X*] were calculated using Equation (9) and are displayed on a linear scale. The lower panel displays a zoom into a region containing fibre crossings between the *corpus callosum* and the corticospinal tract. The dashed‐line box in the top‐left map identifies the high‐*R*_2_ fibres found in the *globus pallidus*

The relaxation and diffusion features from different fibres can be investigated by using Equation (9) to map *R*_2_‐**D** metrics onto the ODF mesh and define orientation resolved means, $\hat{E}\left[X\right]$. The estimated $\hat{E}\left[X\right]$ values are then visualised as colour‐coded ODF glyphs such as the ones displayed in Figure 6, which inform on the correlations between **D** orientation and *R*_2_, *D*_iso_, or $D_{\Delta }^{\;2}$. The displayed ODF maps capture the expected diffusion properties of healthy WM, namely a constant $\hat{E}\left[D_{\Delta }^{2}\right]∼1\times 10^{-9}\;m^{2}s^{-1}$ and a high anisotropy $\hat{E}\left[D_{\Delta }^{2}\right]∼0.7$. The anisotropy metric $\hat{E}\left[D_{\Delta }^{2}\right]$ is found to be unaffected by the presence of fibre crossings (see lower right panel of Figure 6); this is in contrast to the widely used Fractional Anisotropy (FA) metric, which is highly dependent on the degree of orientational order (Basser & Pierpaoli, 1996). Significantly lower $\hat{E}\left[D_{\Delta }^{2}\right]$ values are found at WM–GM interfaces, an observation that can be explained by partial volume effects with GM tissues, which have a lower diffusion anisotropy (see discussion of Figure 3b–d in Section 3.2). Finally, we note that glyphs close to ventricles do not reveal an increased *D*_iso_ or decreased *R*_2_, thus evidencing the successful resolution of signal contributions from CSF.

Focusing on the $\hat{E}\left[R_{2}\right]-\text{coloured}$ ODFs shown in the left side panels of Figure 6, we find a population of fibres with considerably high $\hat{E}\left[R_{2}\right]$ values in the midbrain region (see dashed box in the top left map of Figure 6). The fast‐relaxing ODFs can be attributed to the myelinated axons that traverse the *globus pallidus*, an iron‐rich basal ganglia structure that is characterised by particularly high *R*_2_ values (Hasan, Walimuni, Kramer, & Narayana, 2012; Knight et al., 2015). Not accounting for their significantly different *R*_2_ would then lead to an underestimation of the signal fraction of those high‐*R*_2_ anisotropic components. Moreover, acquiring diffusion‐weighted data measured at a single relatively high *τ*_E_ could even obscure the presence of anisotropic tissues in the *globus pallidus*.

**FIGURE 7:**
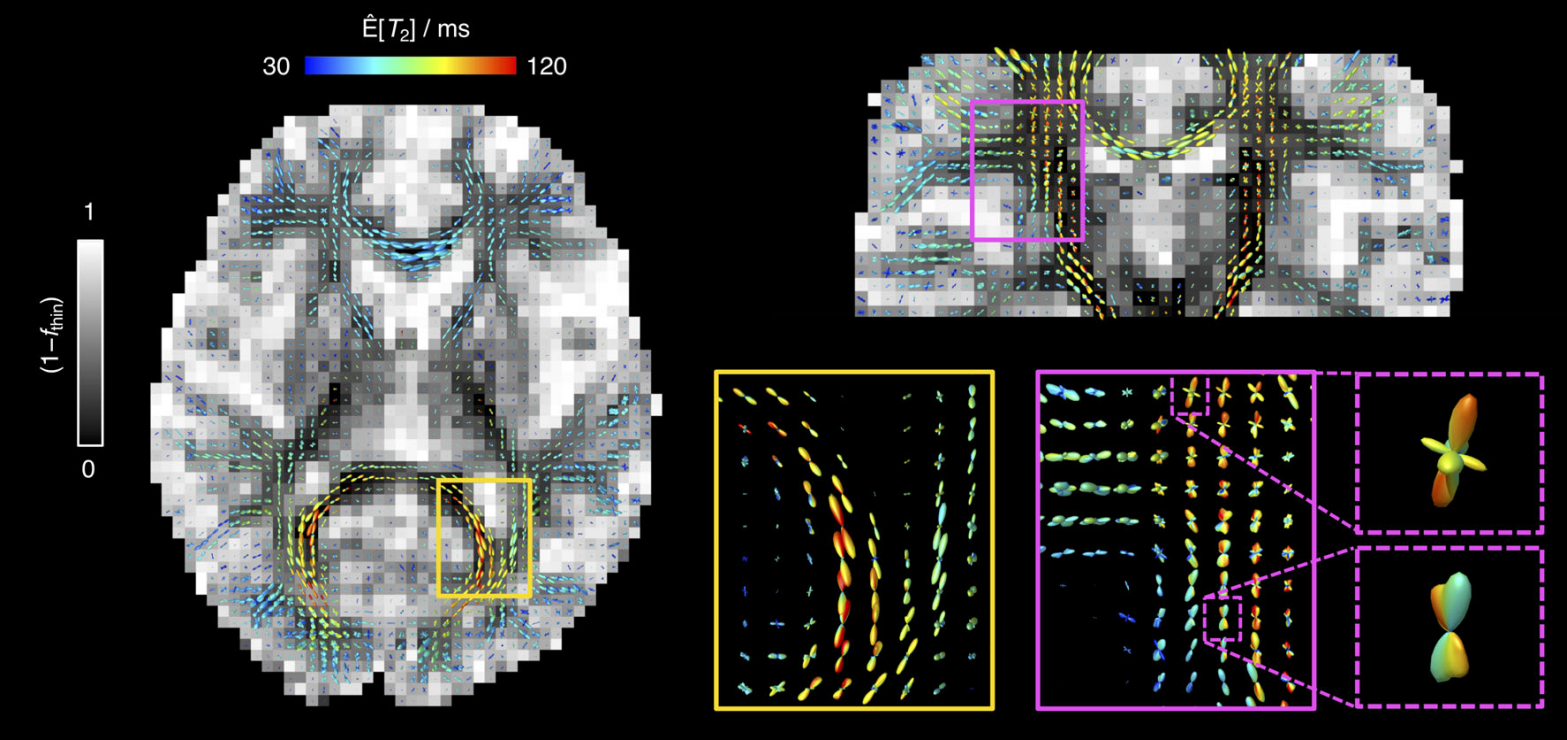
Orientation Distribution Function (ODF) maps coloured according to the orientation‐resolved means of *T*_2_, Ê[*T*_2_]. The Ê[*T*_2_] values are displayed on a linear colour scale. The left and top‐right panels display the sets of ODF glyphs superimposed on a grey‐scaled map showing the signal fractions from the ‘big’ and ‘thick’ bin populations (1 − *f*_thin_) (non‐fibre‐like components). The zoom‐ins in the lower‐right panel offer a more detailed look into selected regions (continuous line boxes) and voxels (dashed line boxes) containing crossing between fibre populations with distinct Ê[*T*_2_]. The observed high‐*T*_2_ components are assigned to the *forceps major* (yellow boxes) and the corticospinal tract (magenta boxes)

Comparing *R*_2_ peak‐specific $\hat{E}\left[R_{2}\right]$ values against their respective *θ* coordinates did not reveal a clear relationship between $\hat{E}\left[R_{2}\right]$ and peak orientation (Figure S2). As detailed in the Supporting Information, the inability to detect a subtle variation of *R*_2_ with varying fibre orientation is attributed to the relatively high uncertainty of the 5D *P*(*R*_2_,**D**) distributions. Despite the fact that no global *R*_2_(*θ*) behaviour could be teased out, the proposed method allowed the detection of relaxation differences between distinct WM tracts. As shown in Figure 7, these differences are best visualised in a *T*_2_ scale spanning a more constrained interval of values than the *R*_2_ scale used in Figure 6. Inspection of Figure 7 reveals that both the CST and the *forceps major* tracts are characterised by considerably $\text{longer}\;\hat{E}\left[T_{2}\right]\;\left(\text{lower}\;\hat{E}\left[R_{2}\right]\right)$ values. These observations are in accordance with the results of Lampinen et al. (2020), where longer *T*_2_ values were consistently found in the CST. The longer *T*_2_ of the CST is also observed in voxels containing fibre crossings, with $\hat{E}\left[T_{2}\right]$ differences being discerned between the ODF lobes corresponding to the CST and the lobes that capture fibre populations from other tracts (see bottom right panels of Figure 7). Moreover, inter‐track *T*_2_ differences can also be observed in the tractograms displayed in Figure S3 of the Supporting Information, where the spatial distribution of high‐*T*_2_ streamlines correlates well with the high‐*T*_2_ ODFs of Figure 7. While the exact mechanisms driving the long *T*_2_ values found in the CST and the *forceps major* are still unclear, it is worth mentioning that these tracts are known to feature higher‐than‐average fractions of large axons (Dell'Acqua et al., 2019), meaning that differences in surface relaxation might play a part in determining the inter‐tract *T*_2_ differences.

While useful for visualisation purposes, the colour‐coded glyphs derived in this work are however impractical for quantifying the dispersion of *R*_2_‐**D** descriptors within a given ODF lobe. For example, the in silico distributions from Figure 2a demonstrate that a single fibre population may comprise a dispersion in *T*_2_ values that cannot be recovered from the summary $\hat{E}\left[T_{2}\right]$ value of the associated ODF peak. Moreover, experimental noise is known to promote a broadening of the recovered distributions (Mitchell et al., 2012), thus amplifying any underlying dispersion and possibly introducing small variations in the *R*_2_‐**D** properties of components within a given lobe. As shown in Figure S4 of the Supporting Information, noise‐induced artefacts may result in spurious low‐amplitude lobes and can affect the mapping of relaxation and diffusion metrics onto the ODF glyphs, creating a colour shading within a single lobe. While substantial degrees of smoothing can be used to alleviate the artefactual shading, such an approach can only offer a partial solution to an effect that originates from uncertainties in the basis *P*(*R*_2_,**D**) solutions.

**FIGURE 8:**
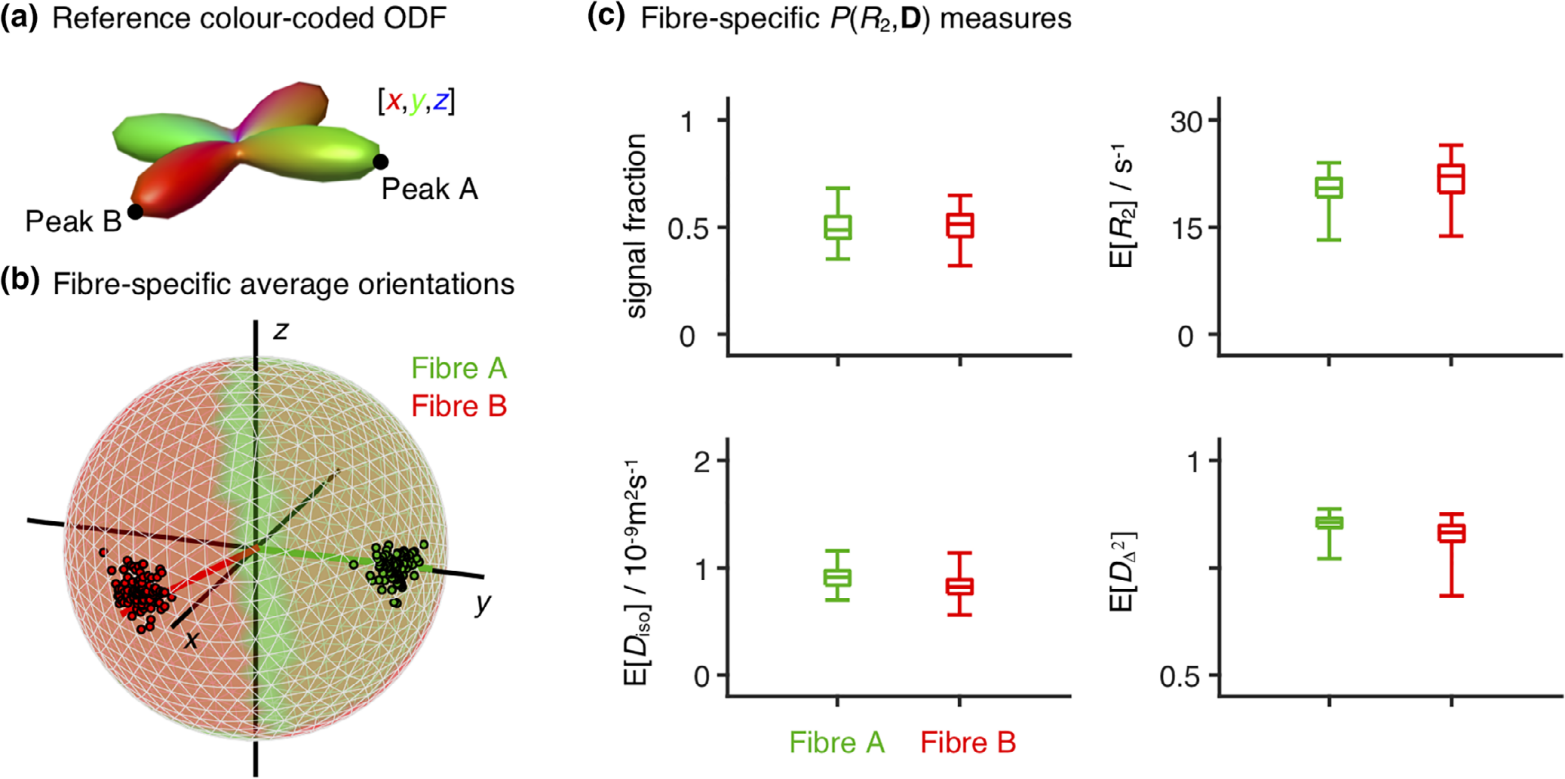
Orientation‐resolved metrics estimated for a two‐fibre‐crossing voxel in the superior longitudinal fasciculus. (a) Orientation Distribution Function (ODF) estimated for the selected voxel. The black points identify the two peaks of the displayed ODF, peaks A and B. (b,c) Fibre‐specific *R*_2_‐**D** metrics. The (*θ*,*ϕ*) orientation space was divided into four quadrants centred on A, B, and their corresponding antipodes; ‘thin’ *R*_2_‐**D** components, $\xi_{n_{b}}^{\text{thin}}$, were then assigned to either fibre population A or fibre population B depending on their (*θ*,*ϕ*) coordinates (e.g., $\xi_{n_{b}}^{\text{thin}}$ components falling into the quadrant centred on peak A, are assigned to fibre population A). For each orientation bin and each bootstrap, we estimate the mean signal fraction, *R*_2_, isotropic diffusivity *D*_iso_, squared normalised diffusion anisotropy $D_{\Delta }^{2}$, and orientation, thus obtaining a set of 96 × 6 scalars: 96 different estimates of six distinct parameters. (b) Ensemble of fibre‐resolved orientations displayed on the unit sphere. The colouring of the sphere identifies the (*θ*,*ϕ*) space assigned to each fibre population. The coloured lines indicate the peak orientation of fibres A (green) and B (red), while the black lines indicate the [*x*,*y*,*z*] coordinates. (c) Boxplots displaying the average and dispersion of the fibre‐resolved signal fractions, *R*_2_,*D*_iso_, and $D_{\Delta }^{2}$. The average was estimated as the median, while dispersion was assessed as the interquartile range. The whiskers identify the maximum and minimum estimated values

To address the limitations identified in the previous paragraph, we suggest using the ODFs and corresponding peaks as a guide to define additional bins in the (*θ*,*ϕ*) space and to subsequently assign the voxel‐wise $\varepsilon_{n_{b}}^{\text{thin}}$ components into the various orientation‐resolved bins. Once the orientation bins have been defined and the $\varepsilon_{n_{b}}^{\text{thin}}$ components assigned, orientation‐specific statistical metrics and uncertainty measures can be estimated by exploring the variability of components within a given (*θ*,*ϕ*)‐bin. An illustration of this procedure is presented in Figure 8 for a voxel comprising two crossing fibres. There, the (*θ*,*ϕ*) ‐ space was divided into four quadrants centred around the extracted ODF peaks; average and dispersion measures were then calculated as the median and interquartile range of the $\varepsilon_{n_{b}}^{\text{thin}}$ components falling within each quadrant. The average fibre‐specific metrics can additionally be used to define a unique colour for its corresponding ODF lobe, thus providing ODF glyphs with a more unambiguous interpretation.

The procedure depicted in Figure 8 showcases the potential of using *P*(*R*_2_,**D**) distributions to extract the average and variance of fibre‐specific metrics. In a preliminary work (Reymbaut et al., 2020), we combine the presented ODF framework with density‐based clustering algorithms (Rodriguez & Laio, 2014) in order to sort $\varepsilon_{n_{b}}^{\text{thin}}$ into different fibre populations and then calculate fibre‐specific statistical metrics from the clustered *P*(*R*_2_,**D**) components.

## 4 CONCLUSION

This work presents analysis protocols to estimate and visualise orientation‐resolved *R*_2_‐**D** metrics in the living human brain. We build on a recently developed 5D relaxation–diffusion correlation framework where sub‐voxel heterogeneity is resolved with nonparametric *P*(*R*_2_,**D**) distributions (de Almeida Martins et al., 2020), and convert the recovered distributions to ODF glyphs informing on the relaxation–diffusion features along different orientations by mapping discrete *P*(*R*_2_,**D**) components to a dense mesh of (*θ*,*ϕ*) bins. Orientationally coloured ODFs estimated in such a way were observed to capture fibre crossings in major WM tracts such as the CC, the CST, or the SLF. Similarly, arrays of $T_{2}-,R_{2}-,D_{\mathrm{iso}}-,\text{and}\;D_{\Delta }^{\;2}-\text{coloured}$ ODF glyphs facilitated a clean and compact visualisation of the *R*_2_‐**D** properties of anisotropic tissues. Maps of relaxation‐coloured ODF also enabled the identification of fast‐relaxing anisotropic components in the *globus pallidus* and the observation of long *T*_2_ times in the CST and the *forceps major*.

The proposed framework relies on 5D *R*_2_‐**D** distributions that provide a clean 3D mapping of the signal contributions from different sub‐voxel tissue environments and allow the estimation of relaxation or diffusion differences between distinct fibre populations. Moreover, the *P*(*R*_2_,**D**) are retrieved from the data without the need to *a priori* fix signal response functions or formulate assumptions about the number of microscopic tissue components. This is in contrast with traditional (Anderson, 2005; Dell'Acqua et al., 2007; Dell'Acqua & Tournier, 2019; Jian & Vemuri, 2007; Tournier et al., 2004; Tournier et al., 2007) or multi‐tissue (Jeurissen et al., 2014) spherical deconvolution approaches, which assume a single response function for WM tissue and do not accommodate microstructural differences across fibres. The caveat is that the proposed method hinges on signal acquisition in a high dimensional space in order to better capture the signal contrast between environments with different MR properties (Topgaard, 2019); a comprehensive sampling of this space in turn introduces acquisition times that are longer than those currently used in spherical deconvolution protocols. However, there is potential to reduce the scan time either by using multi‐band acquisition schemes (Barth, Breuer, Koopmans, Norris, & Poser, 2016) or designing more abbreviated acquisition protocols. Recent advances in nonparametric protocol optimization (Bates, Daducci, & Caruyer, 2019; Song & Xiao, 2020) are expected to facilitate a reduction of the number of required data points while keeping a good performance of the Monte Carlo inversion procedure. Protocol optimization strategies can additionally be used to maximise the angular coverage of the acquisition scheme and hopefully increase the angular resolution of the recovered distributions (Caruyer et al., 2011).

As evidenced by Figure 5 and Figure S3 of the Supporting Information, the information retrieved with the presented methodology can serve as an input for fibre tracking algorithms and used to extract individual WM pathways. If combined with tractometry frameworks (Bells et al., 2011; Chamberland et al., 2019; De Santis et al., 2014; Rheault et al., 2017; Yeatman et al., 2012), the correlations across the *R*_2_‐**D** space would allow a comprehensive inspection of the relaxation and diffusion properties along a given WM tract. Since no universal signal response kernels are assumed, microstructural differences between tracts can be investigated and teased out. This feature is particularly promising for clinical research studies (Fornito, Zalesky, & Breakspear, 2015) where the 5D *R*_2_‐**D** correlation framework could be used to investigate pathology induced changes along specific WM bundles.

## CONFLICT OF INTERESTS

João P. de Almeida Martins, Alexis Reymbaut and Daniel Topgaard declare their status as former employee, employee, and employee/co‐owner, respectively, of the private company Random Walk Imaging AB (Lund, Sweden), which holds patents related to the described method. Filip Szczepankiewicz and Daniel Topgaard are inventors on patents related to the study that are owned by Random Walk Imaging AB. The remaining authors declare no competing interests.

## Supporting information


**Appendix S1** Supporting Information

## Data Availability

The algorithms described in this wok have been incorporated in the multidimensional diffusion MRI toolbox (Nilsson et al., 2018): https://github.com/JoaoPdAMartins/md-dmri. The presented in vivo dataset may be directly requested from the authors.
