# Supplementary material for: Computing and visualising intra‐voxel orientation‐specific relaxation–diffusion features in the human brain
Source: Hum Brain Mapp. 2020 Oct 6;42(2):310–28. doi: 10.1002/hbm.25224 (PMC7776010; doi:10.1002/hbm.25224)
Supplement: Supplementary file 1 — Appendix S1 Supporting Information [file HBM-42-310-s001.pdf]

SUPPORTING INFORMATION

Computing and visualising intra-voxel orientation-specific relaxation-diffusion features in the human brain

João P. de Almeida Martins[1,2], Chantal M. W. Tax[3,4,*], Alexis Reymbaut[1,2], Filip Szczepankiewicz[5,6], Maxime Chamberland[3], Derek K. Jones[3,7], Daniel Topgaard[1,2]*

[1]*Division of Physical Chemistry, Department of Chemistry, Lund University, Lund, Sweden*
[2]*Random Walk Imaging AB, Lund, Sweden*
[3]*Cardiff University Brain Research Imaging Centre (CUBRIC), Cardiff University, Cardiff, United Kingdom*
[4]*University Medical Center Utrecht, Utrecht University, Utrecht, The Netherlands*
[5]*Department of Clinical Sciences, Lund University, Lund, Sweden*
[6]*Harvard Medical School, Boston, MA, United States*
[7]*Mary MacKillop Institute for Health Research, Australian Catholic University, Melbourne, Australia.*

** These authors contributed equally*
Correspondence to: João P. de Almeida Martins (joao.martins@fkem1.lu.se)

S.1 Diffusion-encoding gradient waveforms

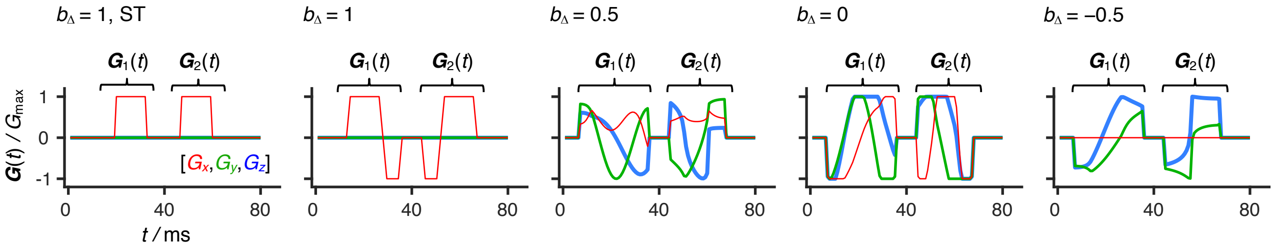

Figure S1. Set of gradient waveforms used in this study. The ST acronym identifies a standard Stejskal-Tanner waveform whose spectral profile (Callaghan & Stepišnik, 1996; Lundell et al., 2019) is distinct from those of the non-monopolar waveforms. The waveforms yielding $b_\Delta = -0.5$, 0, and 0.5 were optimized according to the numerical procedure described in refs. (Sjölund et al., 2015) and (Szczepankiewicz, Westin, & Nilsson, 2019). The displayed waveforms were inserted within a spin-echo sequence with an EPI readout. To clarify the locations of the spin-echo radio-frequency pulses and the EPI block, we divide each waveform in two components: $\boldsymbol{G}_1(t)$ and $\boldsymbol{G}_2(t)$. The 90° pulse is executed at $t = 0$, before the $\boldsymbol{G}_1(t)$ component, while the 180° pulse is applied after a time $t = \tau_E/2$ and is bracketed by the $\boldsymbol{G}_1(t)$ and $\boldsymbol{G}_2(t)$ components. Signal readout starts shortly after the conclusion of $\boldsymbol{G}_2(t)$, with $k = 0$ of the EPI block coinciding with the echo-time τ_E. Relaxation-weighting is achieved by varying τ_E while keeping constant the location of $\boldsymbol{G}_1(t)$ and $\boldsymbol{G}_2(t)$ relative to the 180° pulse.

S.2 Orientation dependence of R_2-D peak-specific metrics

To explore a possible angular dependence of the R_2-**D** metrics from different fibre populations, we computed peak-specific $\hat{\mathrm{E}}[X]$ values and compared them against their respective θ coordinates. The results are displayed in **Figure S2**, where no clear relationship between $\hat{\mathrm{E}}[X]$ and peak orientation is observed for any of the extracted metrics. This observation is in contrast with previous *in vivo* brain MRI studies where a relationship between the R_2 rates of myelinated fibres and their orientation relative to $\boldsymbol{B}_0$ (Gil et al., 2016; Knight, Wood, Couthard, & Kauppinen, 2015; McKinnon & Jensen, 2019; Tax, Kleban, Barakovic, Chamberland, & Jones, 2020) has been reported. In particular, Gil and co-workers (Gil et al., 2016) have estimated an angular variation of $\Delta R_2 \sim 1.5$ s^{-1} for healthy white matter (WM) tissue. **Figure 2A** of the main document shows that the uncertainty of the Monte Carlo analysis procedure can introduce R_2 differences of up to $\Delta R_2 \sim 2.4$ s^{-1} within a single fibre population; this suggests that a subtle R_2 variation is very challenging to resolve with our minimally constrained approach and explains the approximately constant value observed in **Figure S2** for the $\hat{\mathrm{E}}[R_2]$ and $\hat{\mathrm{E}}[T_2]$ metrics. To better assess the minimum R_2 uncertainty of our analysis protocol, we focused on individual CC voxels yielding single-lobe ODFs and computed the interquartile range of mean R_2 values obtained from different bootstrap $\mathcal{E}^{\mathrm{thin}}_{n_\mathrm{b}}$ components. This yielded interquartile ranges of 1.5–2.5 s^{-1} for the various CC voxels, providing further evidence that the R_2 uncertainty of a single fibre population determined through the Monte Carlo inversion is on the same order of magnitude as the R_2 orientational effects reported in ref. (Gil et al., 2016).

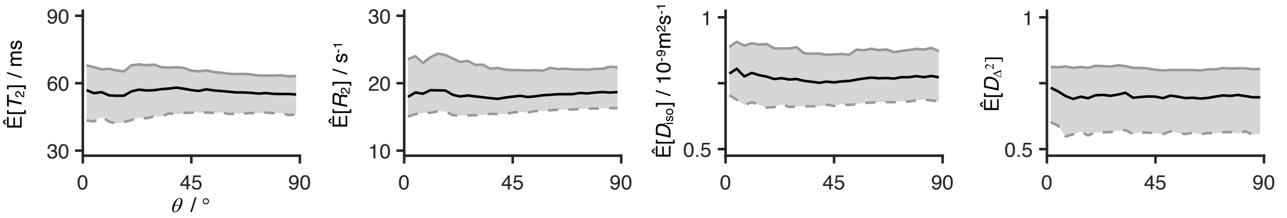

Figure S2. Peak-specific means, $\hat{\mathrm{E}}[X]$, of T_2, R_2, isotropic diffusivity D_{iso}, and squared normalized diffusion anisotropy D_Δ^2 plotted as a function of θ, the polar angle describing the orientation of the various peaks relative to the laboratory frame of reference. The peaks were estimated from local maxima of the smooth ODF, as described in the *Methods* section. The θ angles were sorted into 30 different bins. The solid grey, solid black, and dashed grey lines represent the 75th, 50th (or median), and 25th percentile, respectively, of each angle bin. The shaded grey region illustrates the interquartile range of each angle bin.

S.3 R_2-D coloured tractography

To explore the potential of the suggested framework in teasing out WM pathways with distinct R_2-**D** properties, we implemented a novel visualization approach in FiberNavigator (Chamberland, Whittingstall, Fortin, Mathieu, & Descoteaux, 2014) allowing for the colouring of tracks according to their respective peak amplitudes during real-time tracking. T_2 and D_Δ^2 values were subsequently encoded in the amplitudes of the various peaks, and the new FiberNavigator feature used to map the

two metrics on the derived tracks. The results of this approach are displayed in **Figure S3**, which shows a colour-coded tractogram where T_2 and $D_\Delta{}^2$ values are mapped along each streamline. Future, improved versions of the tractograms displayed in **Figure S3** may be used to investigate differences in the microstructural properties of crossing WM pathways.

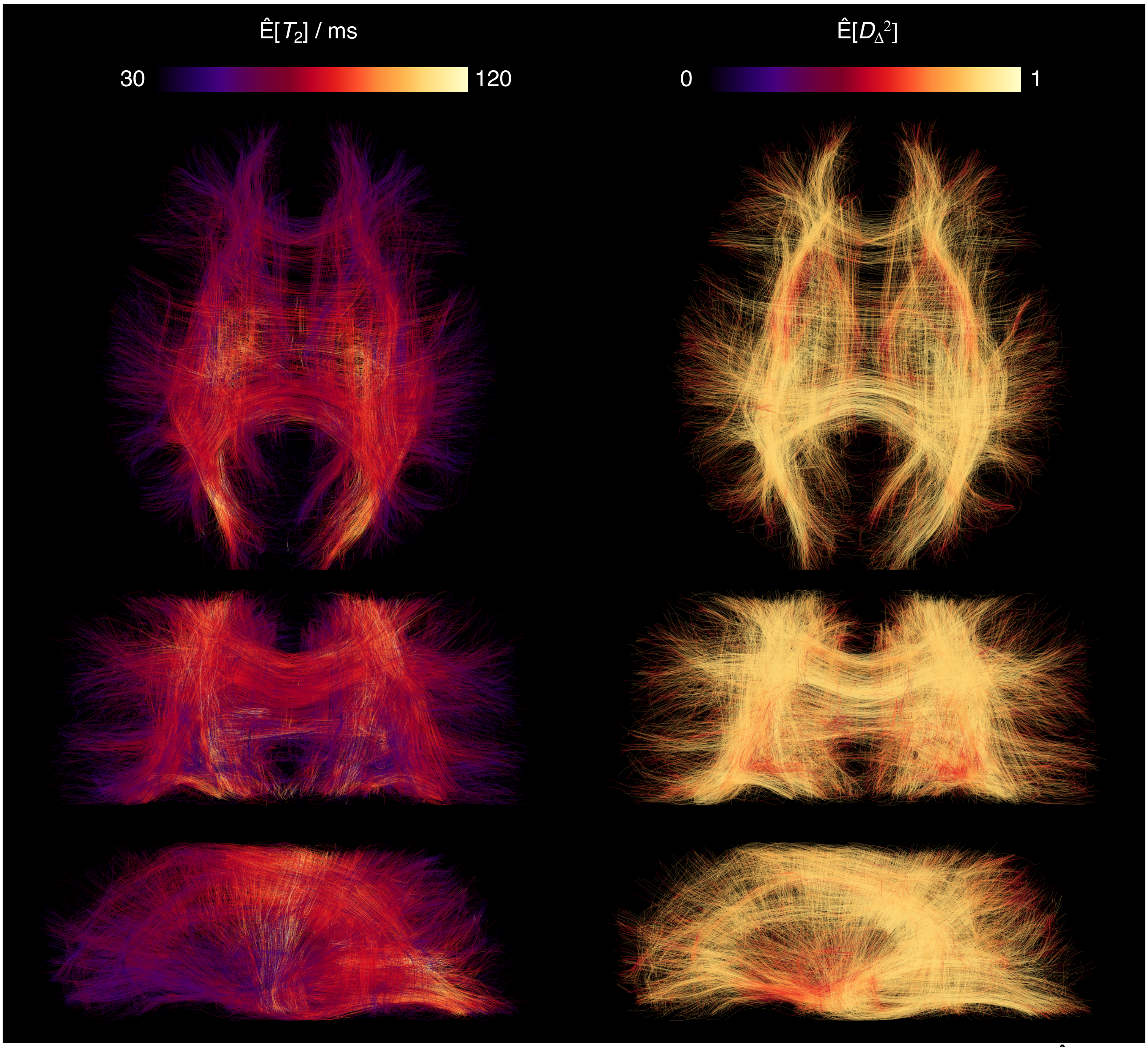

Figure S3. Tractogram with streamlines coloured according to orientation-resolved means, Ê[X], of T_2 (left panel) and squared normalized diffusion anisotropy $D_\Delta{}^2$ (right panel). Top: axial view; Middle: coronal view; Bottom: sagittal view. Global opacity rendering was applied to improve visualisation and remove occlusion.

In accordance with the results from **Figure 7** of the main document, a higher T_2 is visible along streamlines representing the corticospinal tract and the forceps major. Lower $D_\Delta{}^2$ values are found in cortical regions and at the edges of tracts, suggesting a sensitivity of the metric to high degrees of

partial voluming. This observation is consistent with the progressive underestimation of the $\hat{\mathrm{E}}[D_{\Delta}^2]$ peak metric with decreasing fibre signal fractions displayed in **Figure 3D** of the main document. Streamlines with both low T_2 and low D_{Δ}^2 values can be found in the midbrain region – see, for example, the red D_{Δ}^2-coloured streamlines in the middle of the coronal view or the base of the sagittal view – and are likely explained by the presence of deep grey matter tissues with low T_2 times and lower anisotropy (see discussion in the second paragraph of section 3.4 of the main document).

S.4 Effects of noise-induced artefacts on the computed ODFs

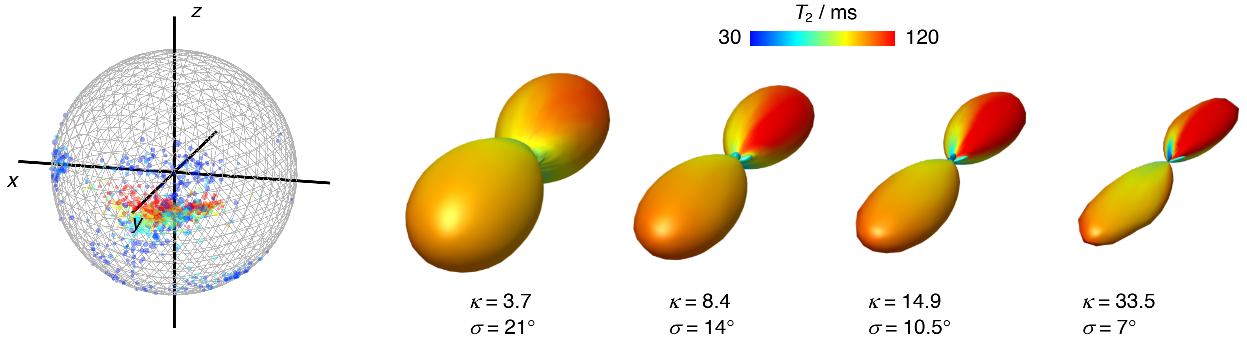

Figure S4. The left panel displays the 'thin' distribution components from a selected R_2-**D** distribution plotted on a unit sphere represented by a 1000-point triangle mesh. The area of each T_2-coloured circle is proportional to the weight of its corresponding component, while the $[x,y,z]$ circle coordinates are defined as $[\cos\phi\sin\theta, \sin\phi\sin\theta, \cos\theta]$, with (θ,ϕ) defining the orientation of each component-wise **D**. The displayed components were retrieved from a single voxel in the forceps major tract. The ODF glyphs are displayed as triangular surface plots on the right side. The four ODF glyphs were computed from the discrete components shown on the left sphere using different angular standard deviation σ of the convolution kernel Eq. (6) of the main document at constant number of triangle vertices. Notice that spurious low-T_2 components give rise to low-amplitude blue-coloured ODF lobes, and that noise-induced differences in the relaxation times of components oriented along y result in a colour shading along the corresponding ODF lobe.

REFERENCES

Callaghan, P. T., & Stepišnik, J. (1996). Generalized analysis of motion using magnetic field gradients. In *Advances in magnetic and optical resonance* (Vol. 19, pp. 325-388): Elsevier.

Chamberland, M., Whittingstall, K., Fortin, D., Mathieu, D., & Descoteaux, M. (2014). Real-time multi-peak tractography for instantaneous connectivity display. *Frontiers in Neuroinformatics, 8*(59).

Gil, R., Khabipova, D., Zwiers, M., Hilbert, T., Kober, T., & Marques, J. P. (2016). An in vivo study of the orientation-dependent and independent components of transverse relaxation rates in white matter. *NMR in Biomedicine, 29*(12), 1780-1790.

Knight, M. J., Wood, B., Couthard, E., & Kauppinen, R. (2015). Anisotropy of spin-echo T2 relaxation by magnetic resonance imaging in the human brain in vivo. *Biomedical Spectroscopy and Imaging, 4*(3), 299-310.

Lundell, H., Nilsson, M., Dyrby, T. B., Parker, G. J. M., Cristinacce, P. L. H., Zhou, F.-L., . . . Lasič, S. (2019). Multidimensional diffusion MRI with spectrally modulated gradients reveals unprecedented microstructural detail. *Scientific Reports, 9*(1), 9026.

McKinnon, E. T., & Jensen, J. H. (2019). Measuring intra-axonal T2 in white matter with direction-averaged diffusion MRI. *Magnetic Resonance in Medicine, 81*(5), 2985-2994.

Sjölund, J., Szczepankiewicz, F., Nilsson, M., Topgaard, D., Westin, C.-F., & Knutsson, H. (2015). Constrained optimization of gradient waveforms for generalized diffusion encoding. *Journal of Magnetic Resonance, 261*, 157-168.

Szczepankiewicz, F., Westin, C.-F., & Nilsson, M. (2019). Maxwell-compensated design of asymmetric gradient waveforms for tensor-valued diffusion encoding. *Magnetic Resonance Medicine, 82*(4), 1424-1437.

Tax, C. M. W., Kleban, E., Barakovic, M., Chamberland, M., & Jones, D. K. (2020). Magnetic Resonance Imaging of T2- and diffusion anisotropy using a tiltable receive coil. In E. Özarslan, T. Schultz, E. Zhang, & A. Fuster (Eds.), *Anisotropy Across Fields and Scales, Mathematics and Visualization*: Springer Nature Switzerland AG.
